# Linker residues regulate the activity and stability of hexokinase 2, a promising anticancer target

**DOI:** 10.1074/jbc.RA120.015293

**Published:** 2020-11-24

**Authors:** Juliana C. Ferreira, Abdul-Rahman Khrbtli, Cameron L. Shetler, Samman Mansoor, Liaqat Ali, Ozge Sensoy, Wael M. Rabeh

**Affiliations:** 1 Science Division, New York University Abu Dhabi, Abu Dhabi, United Arab Emirates; 2 Department of Chemistry, New York University Shanghai, Shanghai, China; 3 The School of Engineering and Natural Sciences, Istanbul Medipol University, Istanbul, Turkey; 4 Core Technology Platforms, New York University Abu Dhabi, Saadiyat Campus, Abu Dhabi, United Arab Emirates; 5 Regenerative and Restorative Medicine Research Center (REMER), Istanbul Medipol University, Istanbul, Turkey; 6 Research Institute for Health Sciences and Technologies (SABITA), İstanbul Medipol University, Istanbul, Turkey

**Keywords:** hexokinase, cancer metabolism, enzyme inactivation, Warburg effect, and anticancer therapeutics, CID, collision-induced dissociation, CTD, C-terminal domain, cv, column volumes, DSC, differential scanning calorimetry, DSF, differential scanning fluorimetry, FL, full-length, G6P, glucose-6-phosphate, *ΔH*_cal_, calorimetric enthalpy, HDX-MS, hydrogen/deuterium exchange mass spectrometry, HIK, heat inactivation kinetics, HK, hexokinase, HK2, hexokinase-2, HKDC1, hexokinase domain containing 1, *K*_ATP_, Michaelis constant for ATP, *K*_Glu_, Michaelis constant for glucose, t_1/2_, half-life, *T*_m_, melting temperature, MD, molecular dynamics, NTD, N-terminal domain, PCA, principal component analysis, PME, particle mesh Ewald, RMSF, root mean-square fluctuations, *v*, initial rate, VDAC, voltage-dependent anion channel

## Abstract

Hexokinase (HK) catalyzes the first step in glucose metabolism, making it an exciting target for the inhibition of tumor initiation and progression due to their elevated glucose metabolism. The upregulation of hexokinase-2 (HK2) in many cancers and its limited expression in normal tissues make it a particularly attractive target for the selective inhibition of cancer growth and the eradication of tumors with limited side effects. The design of such safe and effective anticancer therapeutics requires the development of HK2-specific inhibitors that will not interfere with other HK isozymes. As HK2 is unique among HKs in having a catalytically active N-terminal domain (NTD), we have focused our attention on this region. We previously found that NTD activity is affected by the size of the linker helix-α_13_ that connects the N- and C-terminal domains of HK2. Three nonactive site residues (D447, S449, and K451) at the beginning of the linker helix-α_13_ have been found to regulate the NTD activity of HK2. Mutation of these residues led to increased dynamics, as shown *via* hydrogen deuterium exchange analysis and molecular dynamic simulations. D447A contributed the most to the enhanced dynamics of the NTD, with reduced calorimetric enthalpy of HK2. Similar residues exist in the C-terminal domain (CTD) but are unnecessary for HK1 and HK2 activity. Thus, we postulate these residues serve as a regulatory site for HK2 and may provide new directions for the design of anticancer therapeutics that reduce the rate of glycolysis in cancer through specific inhibition of HK2.

The rate of glucose metabolism is elevated in different types of cancer that primarily utilize aerobic glycolysis, a phenomenon known as the Warburg effect (1, 2). An enhanced glucose metabolic rate is required to meet the increased energy needs and metabolite demands required to support rapid tumor progression (3). HK, the first enzyme in glucose metabolism, catalyzes the irreversible rate-limiting phosphorylation of glucose to glucose-6-phosphate (G6P). In addition to glycolysis, the HK reaction contributes to different pathways, including the tricarboxylic acid cycle and pentose phosphate pathway, for the synthesis of nucleotides, lipids, and amino acids required for rapid tumor growth (3, 4, 5). As an effective regulator of glucose metabolism, HK can be targeted for the inhibition of cancer growth and the development of anticancer therapeutics.

Five human HK isozymes with structurally identical NTD and CTD have been identified, including the new hexokinase domain containing 1 (HKDC1); however, HK4, known as glucokinase, is half the size and contains only a single domain (6, 7, 8, 9). The conserved conformational fold of the NTD and CTD is constructed by small and large subdomains (Fig. 1, *A* and *B*) (6, 7, 10, 11, 12). Each subdomain contains a β-sheet, where the active site is enclosed in a cleft between the β-sheets of the small and large subdomains. D209 and D657 are the catalytic residues positioned at the beginning of the catalytic helix-α_5_ and -α_18_ of the NTD and CTD, respectively. The linker helix-α_13_ is the last secondary structure of the NTD and protrudes from its active site to connect it to the CTD (Fig. 1*D*). The catalytic residues D209 and D657 are required to deprotonate the hydroxyl on C6 of glucose in preparation for its nucleophilic attack on the γ-phosphate of ATP (7, 12, 13, 14).

**Figure 1:**
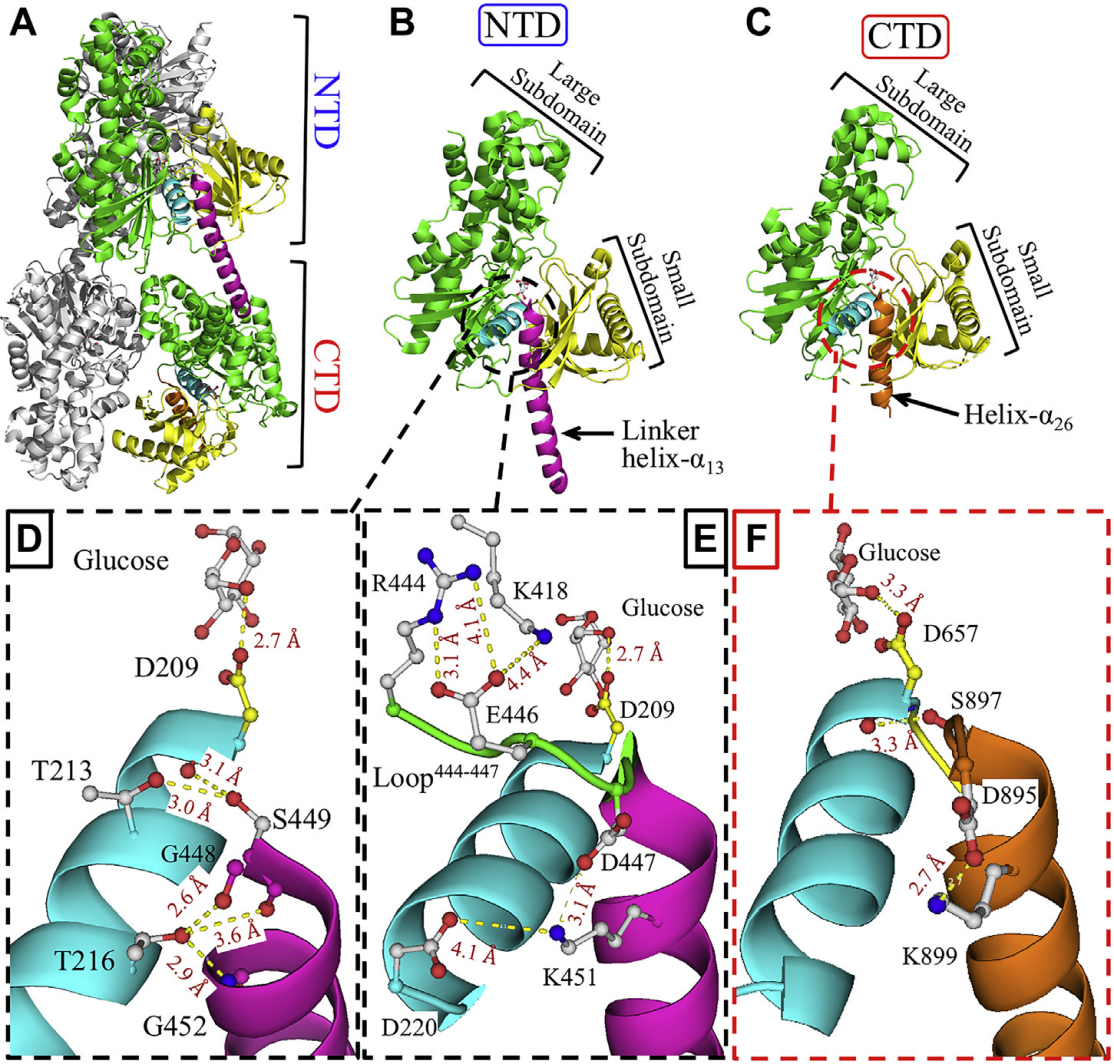
**Overall structural fold and linker helix-α**_**13**_**interaction network of HK2.***A*, cartoon representation of the structural fold of the HK2 homodimer (PDB ID 2NZT). The NTD and CTD of one monomer are colored according to their subdomains and unique secondary structure elements. *B* and *C*, the NTD and CTD of HK2 with the active site located between the large (*green*) and small (*yellow*) subdomains. The catalytic helix-α_5_ and helix-α_18_ (*cyan*) carry the catalytic residues D209 and D657 of the NTD and CTD, respectively. The linker helix-α_13_ (*pink*) connects the NTD and CTD. A small four-turn helix-α_26_ (*orange*) at the end of the CTD is similar to the linker helix-α_13_ of the NTD. *D*, interactions between the linker helix-α_13_ and the catalytic helix-α_5_. Glucose and amino acid side chains (*white*) are shown in ball and stick representation with the D209 side chain in *yellow*. At the beginning of the linker helix-α_13_, S449 forms hydrogen bonds with the side chain of T213 (3.0 Å) and the peptide backbone carbonyl oxygen of D209 (3.1 Å). T216 forms multiple hydrogen bonds with the peptide backbones of G448 (2.6 Å), S449 (3.6 Å), and G452 (2.9 Å). *E*, K451 of linker helix-α_13_ interacts with D447 (3.1 Å) of loop^444−447^ (*green*) and D220 (4.1 Å) on catalytic helix-α_5_. Intramolecular interaction is observed in loop^444−447^ between R444 and E446 (3.1 Å and 4.1 Å). E446 also forms ionic interactions with K418 (4.4 Å) in the large subdomain. *F*, the CTD has a similar network of interactions to the NTD (*E*), where D447, S449, and K451 of the NTD correspond to D895, S897, and K899 in the CTD. The catalytic helix-α_18_ (*cyan*) carries the catalytic residue D657 of the CTD. The figure was prepared using PyMol (Schrodinger LLC).

HK1 is ubiquitously expressed in all mammalian adult tissues and is the main housekeeping isozyme (8). On the other hand, HK2 is upregulated and predominantly expressed in various types of cancers, where it is required to enhance glycolysis for tumor growth and metastasis (5, 15, 16, 17, 18). Silencing HK2 in human hepatocellular carcinoma cells inhibited tumorigenesis and increased apoptosis (5). HK2 was also found to be required for tumor initiation and maintenance in mouse models of lung and breast cancer (17). In addition, HK2 binding to the outer mitochondrial membrane and its interaction with the voltage-dependent anion channel (VDAC) suppress apoptosis and enhance tumor cell survival (19, 20, 21). Though minimally expressed in normal tissues, HK2 is found in embryonic tissues and selectively expressed at the basal level in adipose and muscle adult tissues (5, 15). The biological importance of HK2 for the survival, progression, and chemoresistance of a variety of tumor types and its low abundance in normal tissues make it an attractive target for the development of anticancer therapeutics (17, 18, 22, 23, 24, 25, 26, 27).

Currently, the most commonly used cancer treatment is still chemotherapy, a procedure with high side effects due to its low specificity for cancer cells (27). However, inhibitors of glucose metabolism have been proposed as an effective therapeutic strategy for cancer treatment (5, 27, 28). Due to the highly conserved identity of the active site residues of human HKs, one of the greatest challenges in the design and development of anticancer inhibitors based on the HK reaction is the preferential targeting of HK2 over the other human isozymes (12, 17).

Anticancer therapeutics that target cancer growth will have to specifically inhibit HK2 and avoid interactions with HK in normal tissues. For this reason, successful drug candidates need to bind outside the highly conserved HK active site to avoid the inhibition of all human isozymes. Although HK1 and HK2 share high structural fold similarity, they do exhibit biochemical differences that can be explored for the development of HK2-specific inhibitors. The CTD is catalytically active in all human HKs. Despite its structural fold similarity to the CTD, the NTD is inactive in all human isozymes except HK2. We previously found that the size of the linker helix-α_13_ regulates the activity of the NTD of HK2 (12). When expressed separately from the full-length (FL) enzyme with four of the eight helical turns of the linker helix-α_13_, the NTD variant was inactive. However, increasing the number of helical turns of the linker helix-α_13_ recovered the activity of the NTD variant. The ability of the linker helix-α_13_ to regulate the NTD activity of HK2 makes it an excellent target for the development of specific inhibitors of HK2's active NTD without interacting with or binding to the HK active site.

In this study, we investigated the roles of the linker helix-α_13_ residues on the catalytic activity of the NTD of HK2 to identify a possible regulatory site. Site-directed mutagenesis was used to determine the roles of residues in the linker helix-α_13_ and its network of interactions on the NTD activity of HK2. We identified three residues (D447, S449, and K451) at the beginning of the linker helix-α_13_ that regulate the activity of the NTD of HK2. These residues have been found to maintain the conformational stability around the NTD active site, enhancing the structural stability of the linker helix-α_13_ and its interaction with the catalytic helix-α_5_ of the NTD. CTD of HK1 and that of HK2 contain identical residues to those in the NTD regulatory site, but these residues were unable to control the activity of the CTD. The newly identified NTD regulatory site of HK2 is a promising target for the design of anticancer therapeutics that would reduce the rate of glycolysis in cancer through specific inhibition of the upregulated HK2.

## Results and discussion

Human HK2, similar to isozymes HK1, HK3, and HKDC1, is a homodimer in which each monomer is split into two structurally identical domains, the NTD and CTD (Fig. 1, *A–C*) (10, 11, 12). The overall α/β structural fold of the NTD and CTD is conserved among the mammalian HK family (10, 29, 30, 31, 32, 33), and the active site is located in a cleft between the large and small subdomains. In mammalian HK, the glucose binding site is conserved and includes the catalytic residues D209 and D657 in the NTD and CTD, respectively (12). We previously reported that the linker helix-α_13_ (residues G448–Q478) is important for maintaining the catalytic activity of the NTD (Fig. 1, *A* and *B*), which is composed of a long eight-turn α-helix (12). The linker helix-α_13_ protrudes from the active site at the end of the NTD, thus connecting it to the CTD. It is also perpendicular to the catalytic helix-α_5_ that carries the catalytic residue D209 (Fig. 1*D*). In this study, we investigated the roles of residues of the linker helix-α_13_ on the catalytic activity of the NTD of HK2 to identify a possible regulatory site, which would be targeted in the design of anticancer therapeutics to reduce the rate of glycolysis in cancer through the inhibition of upregulated HK2.

### The catalytic characterization of residues of the linker helix-α_13_ for the identification of a regulatory site in the NTD of HK2

To assess the role of residues of the linker helix-α_13_ in the catalytic activity of the NTD of HK2, site-directed mutagenesis was used to introduce mutants into the linker helix-α_13_ and its network of interactions. Since both the NTD and CTD of HK2 are catalytically active, the enzymatic rate of the NTD was measured in the FL variant in the presence of D657A mutant to catalytically inactive the CTD. All HK2 mutants were expressed and purified using Ni-NTA affinity chromatography followed by size-exclusion chromatography as described before (12). The protein purity was assessed using SDS-PAGE with high purity to enable detailed characterization of the WT and mutant enzymes (Fig. S1).

**Figure 2:**
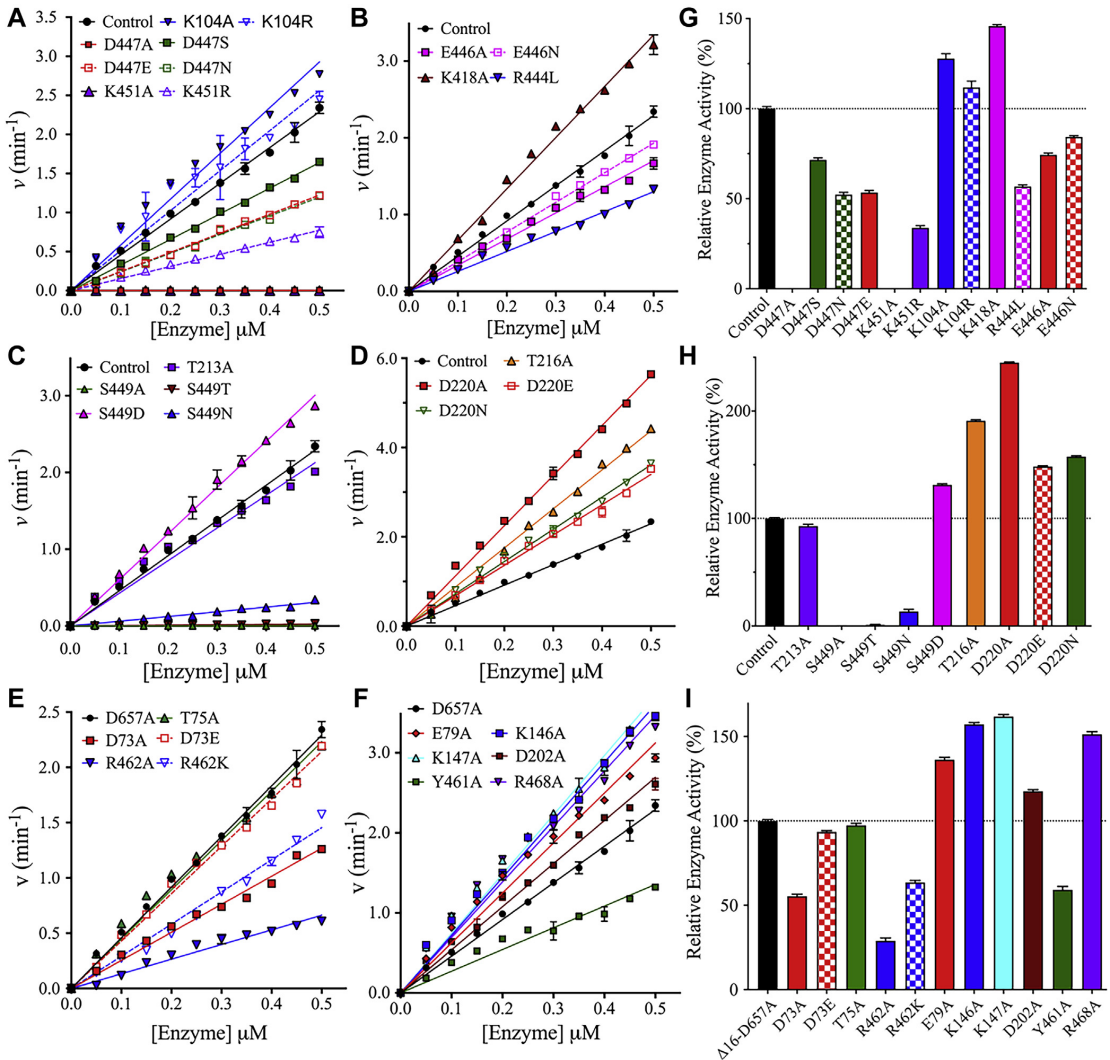
**Enzyme titration analysis of NTD mutants of HK2.***A–F*, the enzymatic rates of the NTD mutants of HK2 were measured at different enzyme concentrations and fixed saturated concentrations of 3 mM for glucose and ATP. The enzymatic rate of the NTD of HK2 was measured in the FL variant only because the CTD was silent in the presence of the D657A mutant of the catalytic residue of the CTD. *G–I*, bar plots of relative enzyme activity determined from the slopes of enzyme rates. Data are the mean ± SD, n = 3.

The enzymatic rate of HK2 mutants was measured at saturated concentrations of glucose and ATP using the coupled G6P dehydrogenase assay. First, the interaction of the linker helix-α_13_ with the catalytic helix-α_5_ and loop^444−447^ of the large subdomain in the NTD was investigated. The last β-strand of the large subdomain extends outward as loop^444−447^ to connect to the linker helix-α_13_, where D447 of loop^444−447^ forms a salt bridge (3.1 Å) with K451 of the linker helix-α_13_ (Fig. 1*E*). K451 is conserved in human HKs, while D447 is partially conserved and replaced by S447 in HK1 and HKDC1 (Fig. S2). The enzyme titration assay showed that D447A and K451A mutants were surprisingly catalytically inactive, where the alanine substitution of each residue was introduced in the presence of D657A in the FL variant (Fig. 2, *A* and *G*). These alanine substitutions at nonactive site residues were able to eliminate the catalytic activity of NTD of HK2. To recover the activity of D447A and K451A mutants, alternative amino acid substitutions were introduced. The NTD activity was partially recovered in the presence of D447E, D447N, D447S, and K451R (Fig. 2, *A* and *G*). The amino acid substitutions D447E and K451R conserved the ionic charges but increased the size of the side chains for these residues, thus decreasing the distance between D447 and K451. The partial recovery of the NTD activity of D447E and K451R mutants indicates that not only the charge but also the distance between D447 and K451 is important in maintaining the activity of the NTD. Therefore, D447 and K451 facilitate strong interactions and mediate a specific distance between the linker helix-α_13_ and loop^444−447^ for optimum activity of the NTD of HK2.

An intramolecular interaction is observed between E446 and R444 (3.1 and 4.1 Å) in loop^444−447^ (Fig. 1*E*), where the NTD enzymatic rate was also reduced by 57% ± 1% and 74% ± 1% in the presence of R444L and E446A mutations, respectively (Fig. 2, *B* and *G*). Interestingly, only HK2 harbors a positively charged residue R444, whereas the corresponding residue is leucine in HK1 and HKDC1, proline in HK3, and E440 in HK4 (Fig. S2). An alternative amino acid substitution, E446N, recovered the enzymatic activity of the NTD. In contrast to D447 and K451, the intramolecular interactions of loop^444−447^ reduced, but did not eliminate the catalytic activity of the NTD of HK2. Furthermore, E446 forms an additional bond with K418 of the large subdomain (4.4 Å), where both residues are conserved in the NTD and CTD of all human HK isozymes (Fig. 1*E*). The K418A mutant increased the activity of the NTD at 146% ± 1% compared with the control (Fig. 2, *B* and *G*).

In the NTD of HK2, the catalytic helix-α_5_ (residues: D209–D220) is a small three-turn α-helix that harbors the catalytic residue D209. The catalytic helix-α_5_ is perpendicular to the linker helix-α_13_, and both are wedged between the two β-sheets of the large and small subdomains (Fig. 1*B*). The linker helix-α_13_ interacts with the catalytic helix-α_5_ at three sites. First, S449 at the beginning of the linker helix-α_13_ interacts with the side chain of T213 (3.0 Å) and peptide backbone of D209 (3.1 Å), both of which are located on the catalytic helix-α_5_ (Fig. 1*D*). Similar to D447A and K451A, the introduction of S449A eliminated the catalytic activity of the NTD of HK2 (Fig. 2, *C* and *H*). Multiple amino acid substitutions were introduced at S449 to evaluate its role in catalysis, where S449N mutant slightly recovered the NTD activity. Interestingly, S449T was inactive, although threonine has the same hydroxyl functional group on its side chain as serine, and S449D slightly increased the NTD activity (Fig. 2*H*). This indicates that not only the hydrogen bonding interaction of S449 but also the size of its side chain is important to maintain the activity of the NTD. To further investigate whether the loss of NTD catalytic activity by S449A is due to interactions with the side chain of T213 or the backbone of D209, the T213A mutant was introduced in the FL variant with D657A, where it did not affect the NTD activity. Therefore, the interaction between T213 and S449 is not important for the NTD activity.

The second point of interaction involves the formation of hydrogen bonds between T216 of the catalytic helix-α_5_ and the peptide backbones of G448 (2.6 Å), S449 (3.6 Å), and G452 (2.9 Å) of the linker helix-α_13_ (Fig. 1*D*). T216A increased the activity of the NTD in the FL variant (Fig. 2, *D* and *H*). Additionally, D220 of the catalytic helix-α_5_ forms a salt bridge (4.1 Å) with K451 of the linker helix-α_13_ (Fig. 2*E*). A distance of ∼4.0 Å has been shown to be optimal for the formation of ionic interactions between the side chains of charged amino acids in proteins (34). The D220A mutant increased the NTD activity by 1.5-fold and eliminated the ionic interactions with K451 (Fig. 2, *D* and *H*). These observations were further confirmed by D220E and D220N, which also increased the NTD activity. Overall, the alanine substitution at T216 and D220 increased the NTD activity, and both are located on the third turn of the catalytic helix-α_5_ opposite D209 (Fig. 1, *D* and *E*). As discussed earlier, the linker helix-α_13_ interactions with the first turn of the catalytic helix-α_5_ harboring the catalytic residue D209 are important in maintaining an active NTD. However, interactions with the third turn of the catalytic helix-α_5_ harboring T216 and D220 hinder the NTD activity.

The third set of interactions is formed between the linker helix-α_13_ and loop^66–77^. The small subdomain is connected to the large subdomain *via* loop^66–77^ and the catalytic helix-α_5_. The R462 of the linker helix-α_13_ interacts with D73 (3.3 Å) and T75 (3.0 Å) of loop^66–77^ (Fig. S3*A*). The NTD activity was affected by D73A and R462A, but no change was observed with T75A (Fig. 2, *E* and *I*). Therefore, the interaction of R462 with D73A is more important than its interaction with T75A. These results were further confirmed when D73E and R462K fully and partially restored the NTD activity, respectively (Fig. 2, *E* and *I*).

The last set of interactions that might influence the NTD activity of HK2 is formed between the second half of the linker helix-α_13_ and the small subdomain. These interactions are facilitated by residues K146, K147, D202, Y461, and R468 with distances >4.4 Å in the crystal structure of HK2 with a closed conformation (Fig. S3*B*) (12). These distances could change in the open conformation, as the subdomains undergo conformational changes to open and close the active site during catalysis, as observed upon glucose binding to HK1 and HK2 (12, 31). Additional internal ionic interactions in the small subdomain are observed between E79 and K147 (3.9 Å) (Fig. S3*B*). The E79A, K146A, K147A, D202A, and R468A substitutions increased the activity of the NTD, whereas Y461A partially reduced its activity (Fig. 1, *F* and *I*). These results show that the interactions of the second half of the linker helix-α_13_ with the small subdomain are not required to maintain the activity of the NTD. Overall, the interactions of residues at the beginning of the linker helix-α_13_ are essential for NTD activity of HK2, whereas interactions at the middle of the linker helix-α_13_ are not.

### Catalytic rate and initial velocity studies of HK2 mutants

**Figure 3:**
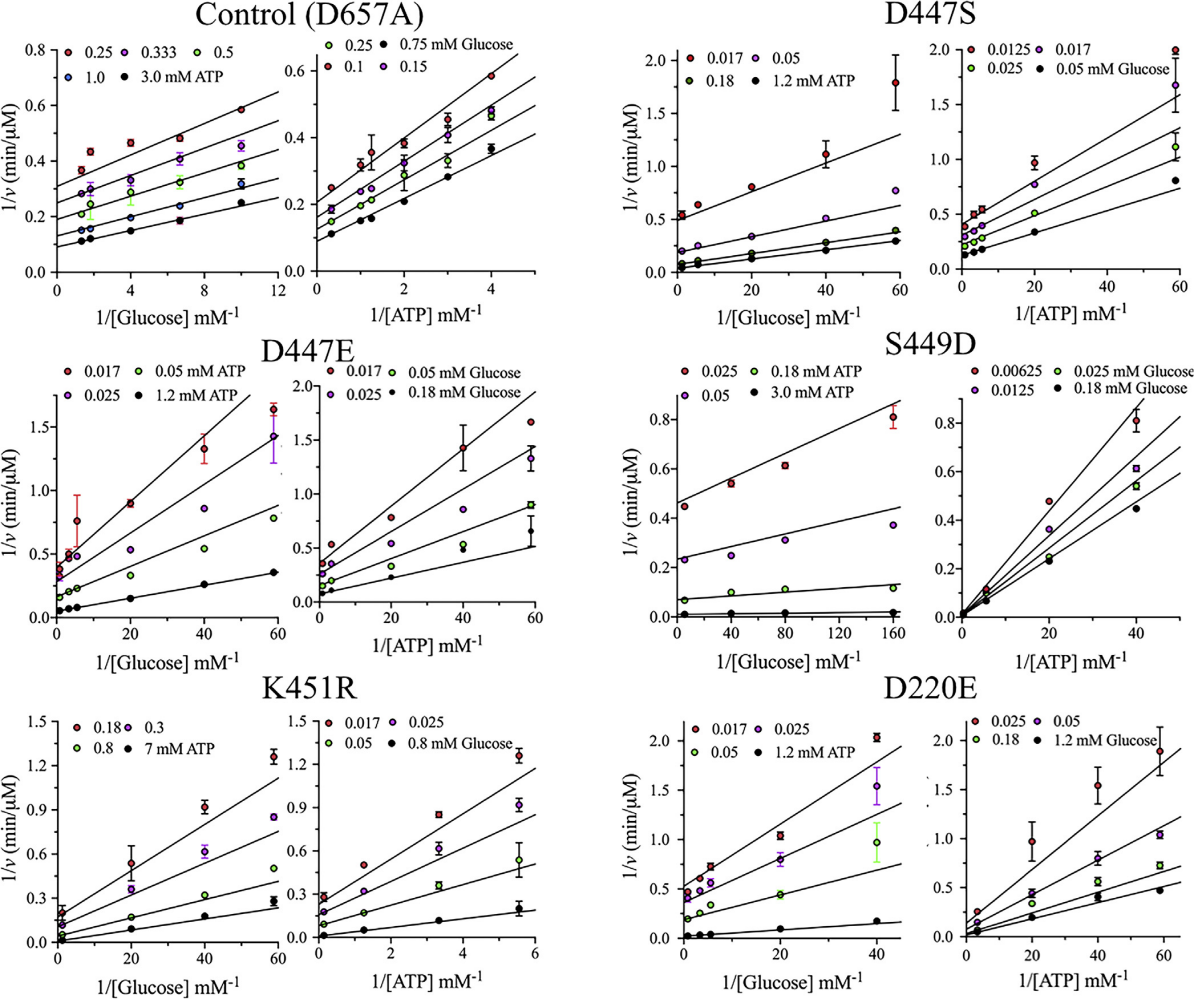
**Double-reciprocal plots of the initial velocity rates of NTD mutants.** The initial velocity rates were determined by varying the glucose concentration at different fixed ATP concentrations. The points on the graphs represent experimental data, and the lines are the theoretical fit of the data to a random Bi–Bi sequential mechanism. The NTD activity of the single mutants D220E, D447S, D447E, S449D, or K451R was measured in the FL variant in the presence of D657A to inactivate the CTD of HK2. The initial velocity rates of the control D658A were also determined. Data are the mean ± SD, n = 3.

Kinetic analysis and initial velocity studies were performed on mutants that affected the NTD activity of HK2. The kinetic studies were conducted on mutants of the NTD regulatory site with several amino acid substitutions of residues D447, S449, and K451. As described earlier, the enzymatic rate of the NTD was directly measured in the FL variant by inactivating the CTD with the D657A mutant. The kinetic parameters were determined from initial velocity studies in the forward direction by varying the glucose concentration at different fixed ATP concentrations. The enzyme rate data were plotted and fitted to random Bi–Bi sequential kinetic mechanism (Fig. 3). The kinetic parameters of all mutants were compared with the control, the D657A mutant. The catalytic rate *V/E*_t_ values of D447S and D447E decreased by twofold, and the Michaelis constant for glucose (*K*_Glu_) slightly decreased for both mutants (Table 1). The Michaelis constant for ATP (*K*_ATP_) decreased by four- and sevenfold for D447S and D447E, respectively, indicating a higher affinity for ATP. Similar to the D447 mutants, a threefold decrease was observed in *V/E*_t_ for K451R, and *K*_ATP_ and *K*_Glu_ increased by four- and sevenfold, respectively (Table 1). Overall, the *V/K*_Glu_*E*_t_ slightly decreased for D447E and D447S mutants, but an 11-fold decrease was observed for K451R. The *V/K*_ATP_*E*_t_ increased by two- and fourfold for D447S and D447E, respectively, but decreased by 18-fold for K451R (Table 1).

**Table 1:** The kinetic parameters of NTD mutants determined in the direction of the formation of G6P at 25 °C and pH 7.5

HK2 variants	*V*/*E*_t_ (s^−1^) fold change	*K*_Glu_ (mM) fold change	*K*_ATP_ (mM) fold change	*V*/*K*_Glu_*E*_t_ (mM^−1^ s^−1^) fold change	*V*/*K*_ATP_*E*_t_ (mM^−1^ s^−1^) fold change
Control D657A	35 ± 1.3	0.19 ± 0.02	0.90 ± 0.07	186 ± 7	39 ± 2
D447S	19 ± 0.4 −1.9	0.15 ± 0.01 −1.3	0.24 ± 0.02 −4	128 ± 3 −1.5	80 ± 2 +2.1
D447E	20 ± 4.5 -1.8	0.12 ± 0.01 −1.6	0.13 ± 0.01 −7	170 ± 38 −1.1	157 ± 35 +4
S449D	41 ± 3 +1.2	0.012 ± 0.002 −16	1.7 ± 0.2 +2	3417 ± 250 +18	24 ± 2 −1.6
K451R	13.4 ± 0.4 −2.6	0.77 ± 0.05 +4.1	6.1 ± 0.3 +7	17.4 ± 1 −11	2.2 ± 0.1 −18
D220A	86 ± 9.2 +2.5	0.21 ± 0.03 +1.1	2.1 ± 0.25 +2.3	410 ± 44 +2.2	41 ± 5 +1.1
D220E	54 ± 1.9 +1.5	0.23 ± 0.02 +1.2	0.63 ± 0.05 −1.4	233 ± 8 +1.3	85 ± 3 +2.2
D73E	34 ± 1.7 +1.0	0.28 ± 0.03 +1.5	1.1 ± 0.14 +1.2	121 ± 6 −1.5	31 ± 2 −1.3
R462K	19 ± 3 −2	0.21 ± 0.02 +1.1	2.0 ± 0.2 +2.2	91 ± 15 −2	10 ± 2 −4

All amino acid substitutions at S449 were catalytically inactive except for S449D. Similar to enzyme titration analysis, the initial velocity studies on S449D revealed that *V/E*_t_ increased by 1.2-fold (Table 1). A twofold increase and 16-fold decrease in *K*_ATP_ and *K*_Glu_, respectively, were observed for S449D with an 18-fold increase in *V/K*_Glu_*E*_t_. The introduction of S449D did not greatly affect the catalytic rate but did increase the glucose affinity of the NTD. Overall, the changes observed in the catalytic rate (*V/E*_t_) and substrates affinities (*K*_Glu_ and *K*_ATP_) support the importance of D447, S449, and K451 in regulating the catalytic activity of the NTD of HK2.

The alanine substitution at D220 that forms a salt bridge with K451 increased the NTD activity of HK2. From initial velocity studies, D220E and D220A showed similar patterns with 1.5- and 2.5-fold increases in *V/E*_t_ compared with the control, respectively (Fig. 3 and Table 1). The substrate binding affinities changed slightly for D220 mutants, except that a twofold increase in *K*_ATP_ was observed for D220A. These results again suggest that the interactions between D220 and K451 hinder the NTD activity of HK2. Finally, to investigate the importance of the interactions between loop^66–77^ and the linker helix-α_13_, initial velocity studies were conducted on D73E and R462K. The kinetic parameters of D73E were similar to those of the control with a slight increase in *K*_Glu_ and *K*_ATP_, and R462K decreased *V/E*_t_ by twofold with no change in *K*_Glu_ and a twofold increase in *K*_ATP_ (Table 1). Consistent with the enzyme titration analysis, D73 and K462 do not play major roles in controlling the NTD activity of HK2.

### Thermodynamic stabilities and half-life characterization of residues in the NTD regulatory site of HK2

**Figure 4:**
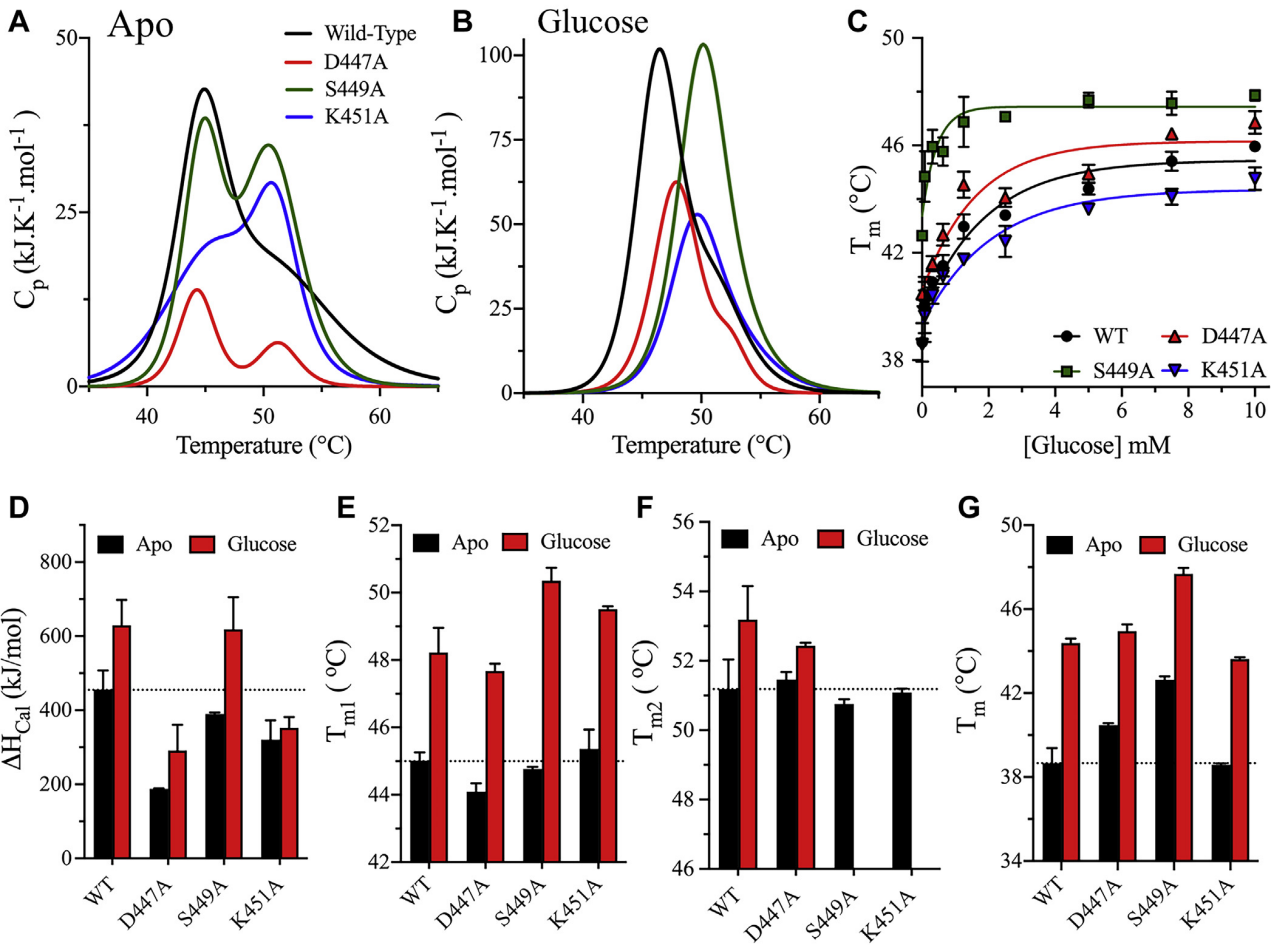
**Thermodynamic stability analysis of NTD mutants.***A* and *B*, the DSC thermograms of FL variants of WT (*black*), D447A (*red*), S449A (*green*), and K451A (*blue*) in the absence (*A*) or presence (*B*) of 5 mM glucose. The DSC scans were corrected for the buffer baseline, and the data were fitted to one- or two-state transitions. The thermograms have been baseline corrected and deconvoluted using the Nano-Analyze software package from TA instruments. *C*, DSF analysis of FL variants for the WT and NTD mutants of HK2 at different glucose concentrations. SYPRO Orange was used as the reporter dye for the determination of T_m_ at different glucose concentrations. *D–F*, bar plots of the DSC parameters *ΔH*_cal_, *T*_m1_, and *T*_m2_ in the absence (*black*) or presence (*red*) of 5 mM glucose for the WT and NTD mutants. The thermograms of S449A and K451A in the presence of glucose were deconvoluted as a one-state transition; therefore, the T_m2_ values in the presence of glucose were not included for S449A and K451A. *G*, bar plots of T_m_ determined from DSF analysis in the absence (*black*) or presence (*red*) of 5 mM glucose. Data are the mean ± SD, n = 3.

Differential scanning calorimetry (DSC) and differential scanning fluorimetry (DSF) were used to determine the thermodynamic stabilities of HK2 mutants in the NTD regulatory site (Fig. 4). Thermodynamic measurements were performed on the FL variants of the single mutants D447A, S449A, and K451A in the absence or presence of glucose. Previously, we reported an increase in the thermodynamic stability of HK2 variants upon glucose binding (12). From DSC analysis, all thermal melts of HK2 were irreversible with a second heating scan included for each run as outlined in the methods section. The HK2 thermograms showed single or two-state transitions, where the melting temperature (*T*_m_) was calculated at the apex of the melting peak, and the calorimetric enthalpy (*ΔH*_cal_) was determined from the area under the thermographic peak. Similar to the WT enzyme, the DSC thermograms of the mutants produced a two-state transition in the apo-state (Fig. 4*A*). The addition of glucose increased the thermodynamic stability of WT and mutants, where the thermograms of S449A and K451A shifted to a single transition and those of WT and D447A yielded a small shoulder for the second transition (Fig. 4*B*). The addition of glucose increased T_m1_ between 3 and 6 °C and *ΔH*_cal_ was higher for WT and S449A at 174 ± 8 and 274 ± 25 kJ/mol compared with the marginal increases of 32 ± 11 and 64 ± 12 kJ/mol for K451A and D447A, respectively (Fig. 4, *D* and *E*). The effect of glucose on T_m2_ was marginal with an increase of less than 2 °C for WT and D447A (Fig. 4*F*). Therefore, D447A or K451A compromised the thermodynamic stabilization effect observed upon glucose binding. We reported a similar observation previously, where glucose did not enhance the enthalpy of D209A, the catalytic residue in the NTD of HK2 (12). The decrease in *ΔH*_cal_ may result from the catalytic inactivation of the NTD of HK2, as mutants that exhibited a decrease in *ΔH*_cal_ possessed an inactive NTD.

In addition to DSC analysis, the T_m_ of HK2 mutants was determined by DSF from the global thermal unfolding of FL variants in the presence of a reporter dye, SYPRO Orange. In the absence of glucose, the T_m_ of S449A (42.6 ± 0.2 °C) was higher than those of WT (38.3 ± 0.9 °C), D447A (40.5 ± 0.1 °C) and K451A (38.6 ± 0.1 °C) (Fig. 4, *C* and *G*). Similar to DSC, the addition of glucose increased the T_m_ of WT and mutants by 5–6 °C, and S449A reached glucose saturation faster than the WT, D447A, and K451A. The T_m_ values reported here by DSF were on average <7 °C lower than T_m1_ estimated from DSC. The difference in the T_m_ values is expected due to the nature of the two techniques, with DSC measuring the molar heat capacity of the protein sample versus DSF utilizing a fluorescence probe, which binds to hydrophobic patches on the protein sample (35).

Another thermodynamic property tested here is the heat inactivation kinetics (HIK) to reveal the relation between the thermal stability and the time-dependent catalytic activity of HK2 mutants. The HIK was measured from the time dependence of the residual catalytic activity of NTD mutants incubated at 37 °C in the absence or presence of glucose. In the apo-state, the decrease in the percentage of relative enzyme activity was the fastest for the WT with the shortest half-life (t_1/2_) of 5 min (Fig. S4*A*). The D447A and K451A mutants possessed higher thermal kinetic stabilities than WT with t_1/2_ values of 8 and 16 min, respectively. However, the D209A and S449A mutants showed the highest kinetic thermal stability with similar inactivation rates in the absence or presence of glucose. The addition of glucose increased the thermal kinetic stabilities of WT, D447A, and K451A with inactivation rates similar to those of D209A and S449A (Fig. S4*B*). The D209A mutant was introduced as a control for mutants with an inactive NTD. Unlike the WT, all mutants analyzed in this study possessed an inactive NTD. The increased kinetic thermal stabilities of the mutants could result from their inactive NTD, as similar results were obtained with the D209A mutant containing an inactive NTD.

### Hydrogen/deuterium exchange mass spectrometry (HDX-MS) to determine the conformational dynamics of mutants eliminated the catalytic activity of the NTD of HK2

We investigated the roles of D447, S449, and K451 in the activity of the NTD, where the alanine substitutions of these residues were sufficient to eliminate the catalytic activity of the NTD of HK2. It was not clear how these residues distant from the active site regulated the NTD activity. To investigate their regulatory mechanisms, HDX-MS was used to compare the dynamics and conformational fold differences between the WT and mutants that demolished the NTD activity of HK2. The deuterium incorporation rate was measured in the absence or presence of glucose to evaluate the structural fold differences of the WT and mutants in the open (ligand-free) and closed (glucose-bound) states. To the best of our knowledge, there is no crystal structure of the open apo-state of mammalian HKs, as all available crystal structures are in complex with ligands, *e.g.,* glucose and G6P (10, 11, 12, 13, 29, 31, 32). Therefore, this HDX-MS analysis represents the first study of conformational dynamics of the apo-state of any mammalian HK.

The HDX-MS measurement of the NTD of HK2 was conducted in two variants, the FL and NTD that is expressed as a separate domain. The NTD or FL variants were incubated in a deuterated buffer at time intervals of 1 min, 6 min, 1 h, and 3 h in the absence or presence of 5 mM glucose. The deuterium incorporation was measured by Q-TOF MS (Impact II, Bruker Daltonic) with a resolution >50,000. The higher charge states (charge greater than +1) with the highest peak intensity were used to determine the deuterium incorporation for each peptide acquired from the protease column. To facilitate differences in protein dynamics between the WT and mutants and to enhance the HDX rates, the deuterium exchange temperature was increased from 25 °C to 37 °C. The temperature increase enhanced the deuterium incorporation rates for the WT and mutants with similar sequence coverage. The number of peptides was 266 and 415 for the NTD and FL variants, respectively, with >95% protein sequence coverage. The peptide sequence maps of the WT and mutant enzymes were identical in the absence or presence of glucose with variations at the linker helix-α_13_ region between the FL and NTD variants of HK2. Due to the high structural identity and sequence similarity of the NTD and CTD, the catalytic helices of both domains share identical amino acid sequences represented by residues 204–218 and 652–666 (VAVVNDTVGTMMTCG), respectively. Consequently, amino acid sequence assignments of the catalytic helices to NTD or CTD in the FL variant were not possible. Therefore, D657A, the catalytic residue mutant of CTD, was used as the control for the FL variant in the HDX-MS experiment to enable amino acid sequence assignment of the catalytic helices to the NTD or CTD of HK2.

**Figure 5:**
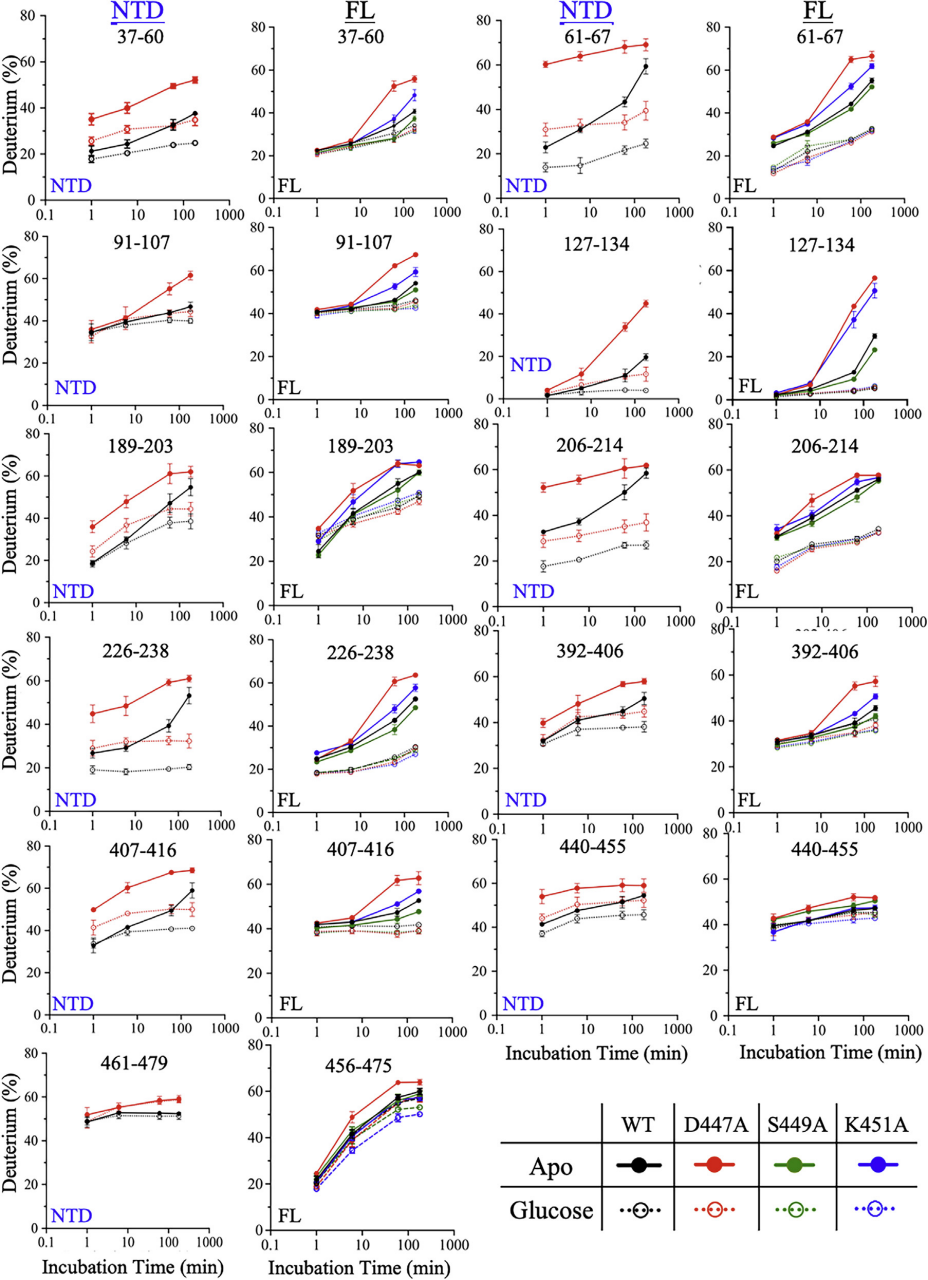
**Comparative HDX kinetics of NTD mutants.** The percentage of deuterium exchange for the NTD mutants was determined as a function of exchange time at 37 °C. The HDX-MS measurements were conducted on the NTD or FL variants. The HDX traces of the representative peptides showed different deuterium exchange rates in the absence (=) or presence (ο) of 5 mM glucose for the WT (*black*), D447A (*red*), S449A (*green*), and K451A (*blue*). Lines through points are shown only as guides. Data are the mean ± SD of 6–8 and 3–6 measurements for the apo and glucose-bound states, respectively.

The time-dependent HDX-MS measurements identified 11 nonoverlapping peptides of the NTD with altered dynamics, modulated by either glucose addition or mutations (Fig. 5). In the apo-state, D447A and K451A increased the deuterium incorporation rates for all peptides compared with the WT, while S449A had similar or reduced deuteration compared with the WT except for residues 440–455 (Fig. 5). The increased dynamics of D447A and K451A were centered on residues 206–214, which represents the first half of the catalytic helix-⍺_5_ (residues 209–220). The deuterium incorporation at residues 206–214 in the FL variant at 1 h increased by 7% and 4% for D447A and K451A, respectively. In addition to the FL variant, HDX-MS analysis was conducted on the NTD variant expressed separately from the FL enzyme, where the WT enzyme was used as a control. In the NTD variant, D447A increased the deuteration of the catalytic helix-⍺_5_ by 16% with only 6 min of incubation. On the other hand, deuterium incorporation of residues 206–214 at 1 h decreased (∼24%) upon the addition of glucose for WT and mutants in the NTD and FL variants (Fig. S5). The decrease in dynamics in the catalytic helix-⍺_5_ upon the addition of glucose is important to prepare the enzyme for catalysis, as slower motions of active site residues facilitate the alignment of substrates for catalysis. The inactivation of the NTD by D447A may result from increased dynamics of the catalytic helix-⍺_5_, where improper alignment of D209 with the C6-hydroxyl of glucose prevents its deprotonation and nucleophilic attack on the γ-phosphate of ATP.

**Figure 6:**
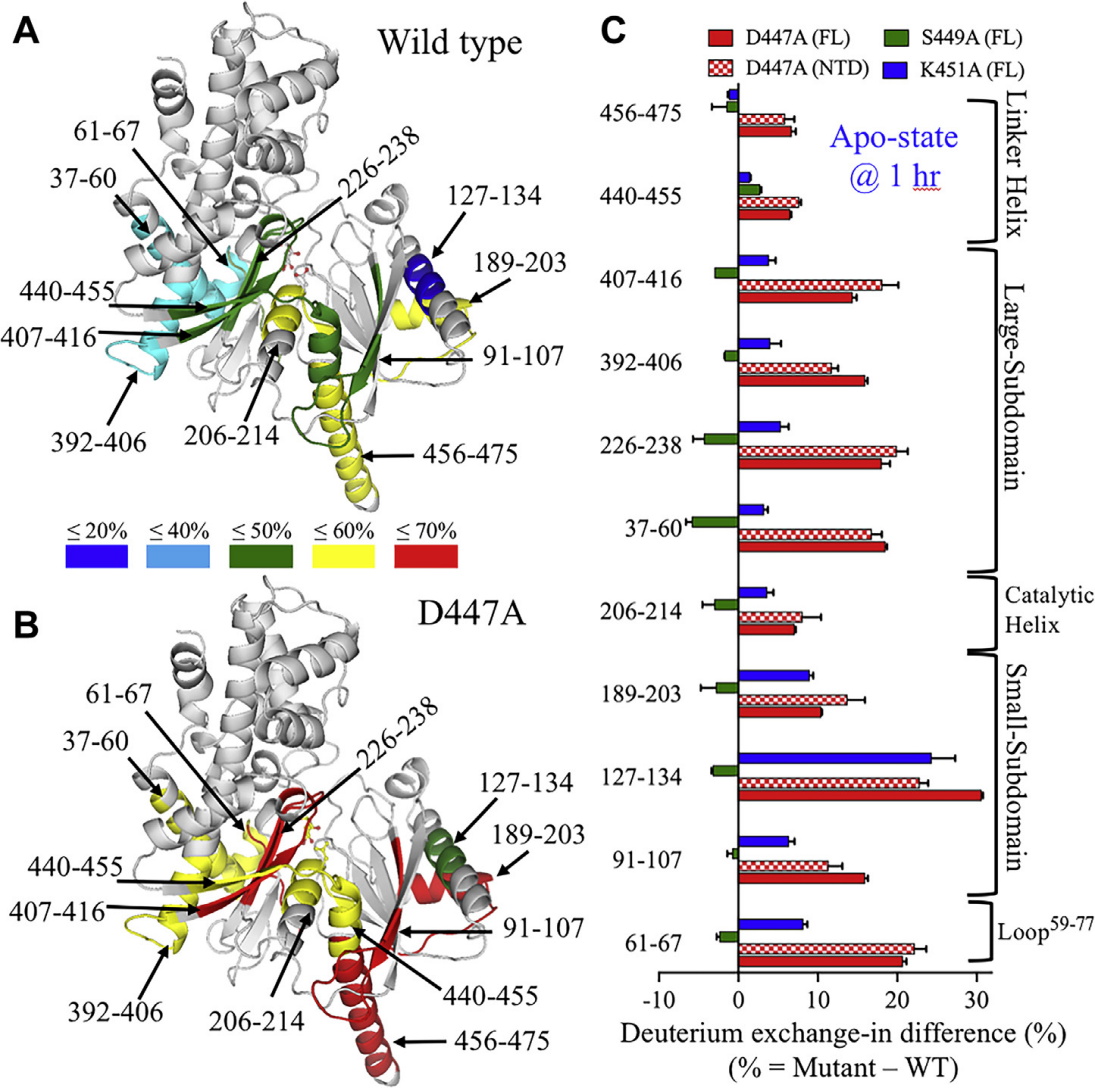
**Comparison of the percentage of HDX for the NTD of WT and D447A.** Graphical presentations of the NTD of HK2 for WT (*A*) and D447A (*B*). Mapping the HDX data of the FL variant at 1 h of the apo-state onto the structure of HK2 (PDB ID 2NZT). The color-coded structures reflect the percentage of HDX, with *blue* being least labeled and *red* most labeled, for peptides with altered HDX exchange profile upon the introduction of D447A. The HDX profile of D447A is shown here, as it exhibits the most deuterium incorporation compared with the other mutants. This figure was prepared using PyMol (Schrodinger LLC). *C*, bar plot of the percent increase in deuterium exchange in the apo-state at 1 h, calculated by subtracting the exchange of WT from that of the mutants. The color code is D447A (*red*), S449A (*green*), and K451A (*blue*) in the FL (*solid bars*) and NTD (*checkered bars*) variants.

Using the percentage of deuterium incorporation data at the 1 h time point for apo-WT and D447A in the FL variant, we mapped the regions that showed higher exchange rates onto the crystal structure of the NTD of HK2 (Fig. 6, *A* and *B*). The graphical presentation of the HDX-MS reveals increased conformational flexibility on regions surrounding the catalytic helix-⍺_5_ of D447A compared with the WT. To evaluate the effect of mutants on the dynamics of HK2, the difference in the percentage of deuterium exchange was calculated by subtracting the HDX percentage of WT from the mutant (Fig. 6*C*). In the apo-state, the D447A mutation increased the dynamics of residues 226–238 and 407–416 of the large subdomain by 18% and 14% in the FL variant and 20% and 18% in the NTD variant, respectively. K451A increased the dynamics by ∼5%, but S449A decreased it by ∼4%. In the large subdomain, three peptides, 226–238, 407–416, and 440–455, are part of the five-stranded β-sheet of the large subdomain that is positioned between the catalytic helix-⍺_5_ on one side and two α-helices (residues 37–60 and 392–406) on the other side. D447A increased the deuteration of residues 37–60 and 392–406 by 18% and 16% in the FL variant and by 17% and 12% in the NTD variant, respectively. While K451A increased the HDX of these peptides by ∼4%, and S449A decreased it by 6% and 2%, respectively. Overall, the four peptides representing the large subdomain showed enhanced dynamics in regions adjacent to the catalytic helix-⍺_5_ in the presence of mutants D447A and K451A (Fig. 6*C*).

In the apo-state, the dynamics of loop^59–77^ (peptide 61–67) that connects the C-end of the large subdomain with the N-end of the small subdomain increased by 21% for D447A in both the NTD and FL variants and by 8% for K451A in the FL variant (Fig. 6, *A*–*C*). In contrast, the dynamics of loop^59–77^ decreased by 2% in the S449A mutant. Similarly, in the small subdomain, D447A increased the deuterium incorporation for peptides 91–107, 127–137, and 189–203 by 11–23% and 10–30% in the NTD and FL variants, respectively. Peptides 91–107 correspond to the first two strands of the five-stranded β-sheet in the small subdomain that is adjacent to linker helix-α_13_ on one side and two α-helices (peptides 127–137 and 189–203) on the other side. However, the deuterium incorporation for these three peptides decreased by <3% in S449A.

Peptides 440–455 are important because they form multiple secondary structures, including the last strand of the β-sheet in the large subdomain, loop^444−447^, and the first turn of the linker helix-α_13_. The deuterium incorporation of peptides 440–455 is one of the highest among the NTD peptides presented here with 40% HDX for the control after 1 min of incubation of the NTD and FL variants in the absence or presence of glucose. The deuteration of peptides 440–455 increased by ∼7% for D447A in the NTD and FL variants and by ∼3% for S449A and K451A in the FL variant. Interestingly, S449A decreased the dynamics of all NTD peptides except peptides 440-455. The side chain of S449 forms a hydrogen bond with the backbone carbonyl oxygen of the catalytic residue D209. The enhanced dynamics of peptides 440–455 would reduce the structural stability of the catalytic helix-⍺_5_ and the alignment of D209 with glucose for catalysis, which explains the ability of the nonactive site residues D447A, S449A, and K451A to eliminate the catalytic activity of the NTD of HK2 due to increased conformational dynamics around the active site.

Finally, the pepsin digestion of the linker helix-α_13_ was different in the NTD variant (residues 461–479) and the FL variant (residues 456–475). In the apo-state and at the 1 h time point, D447A was the only mutant that increased the deuteration of the linker helix-α_13_ compared with the WT by 6% and 7% in the NTD and FL variants, respectively, and S449A and K451A did not alter its deuteration (Fig. 6*C*). However, in the glucose-bound state and at the 1 h time point, D447A did not alter the deuteration of the linker helix-α_13_ compared with the WT, but S449A and K451A reduced its deuteration by 3% and 6%, respectively (Fig. S5). Overall, the deuterium incorporation rate of the linker helix-α_13_ in the FL variant is high compared with that of other peptides observed here, with time-dependent deuterium labeling averaging 21% (1 min), 41% (6 min), and 56% (1 h and 3 h) for the control and mutants in the apo and glucose-bound states (Fig. 5). In the NTD variant, the linker helix-α_13_ deuteration was 50–55% in the apo and glucose-bound states for the WT and D447A. The high dynamics of the long linker helix-α_13_ may be related to its role in the NTD catalysis, where opening and closing of the NTD active site are accompanied by conformational changes in the linker helix-α_13_ (12). In addition, D447A further enhanced the dynamics of the linker helix-α_13_, which could reduce the conformational stability of the NTD active site, enabling conformational perturbation to compromise the ability of the NTD to catalyze a reaction.

### The catalytic activity of the CTD mutants of HK1 and HK2 for cancer drug specificity

HK2 is overexpressed in various types of cancers but limited in normal adult tissues (17), as HK1 is the dominantly expressed human isozyme in normal cells (8). In addition to its role in anaerobic glycolysis, HK2 is necessary for chemoresistance and for tumor initiation and maintenance in many types of cancers (17, 25, 36). Therefore, HK2 is an attractive candidate for the development of anticancer therapeutics; however, the challenge is to achieve the specific inhibition of glycolysis in cancer cells without affecting normal tissues. This can be accomplished if HK2 can be specifically inhibited without affecting other HKs, *e.g.,* HK1. The human HK isozymes share a highly conserved structural fold and active site residues making the specific inhibition of HK2 a challenge.

Here, the newly identified NTD regulatory site of HK2 is a promising target for the specific inhibition of glycolysis in cancer. Since the NTD is naturally inactive in all human HKs except HK2, the design of anticancer drugs that inhibit the NTD of HKs without affecting CTD activity would enable the development of specific anticancer therapeutics. Due to the high degree of structural similarity between the NTD and CTD of human HKs, the D895 and K899 residues of the CTD are identical to the D447 and K451 residues, of the NTD regulatory site (Fig. 1, *D* and *E*). To validate their roles in the activity of the CTD, site-directed mutagenesis analysis was conducted on D895 and K899 in both the HK1 and HK2 isozymes. To measure the CTD activity in the FL variant, D209A was included with HK2 mutants to inactivate its NTD but was not needed for the HK1 mutants, as HK1 contains a naturally inactive NTD. The enzyme titration analysis of the D895A mutant yielded similar CTD activity to the controls for both HK1 and HK2 (Fig. S6, *A–C*). The K899A mutant retained 68 ± 2% and 91 ± 2% of the CTD activity for HK1 and HK2, respectively. Therefore, the NTD regulatory site is a good candidate target for the specific inhibition of HK2 with limited effects on the CTD of other human HKs to achieve the specific inhibition of glycolysis in different cancer types with upregulated HK2 levels.

In summary, the ionic interactions between D895 and K899 are conserved in the CTD of all human HK isozymes (Fig. S2). However, in the NTD, K451 forms a salt bridge with D447 of HK2 and HK3 and E442 of HK4, but K451 forms hydrogen bonds with S447 of HK1 and HKDC1. In the FL variant, D447 and K451 were important for the NTD activity, but D895 and K899 did not affect or partially reduced the CTD activity. One of the main structural differences between the two HK domains lies in the linker helix-α_13_ and helix-α_26_ at the ends of the NTD and CTD, respectively (Fig. 1, *A*–*C*). The linker helix-α_13_ connects the NTD to the CTD, but helix-α_26_ is free and not associated with any structure. To further investigate whether the NTD inactivation by D447 mutants is due to restriction of the linker helix-α_13_ movement in the FL variant, the activity of the D447A and D447S mutants was measured in the NTD variant. The D447A mutant that disrupted the interactions with K451 possessed only 14 ± 1% of the WT activity in the NTD variant (Fig. S6, *D* and *E*), where in the FL variant it was inactive. Nevertheless, the D447S mutant, which can form a hydrogen bond with K451, restored 55 ± 3% of the WT activity in the NTD variant compared with 72 ± 1% of the control activity in the FL variant. Therefore, restricting the linker helix-α_13_ movement in the FL variant does not have major contribution to NTD inactivation, but the D447 bonding interactions with K451 are important in maintaining the NTD activity of HK2.

### Molecular dynamics (MD) simulations and comparison of structural stability of the NTD of HK2 for WT and D447A mutant

**Figure 7:**
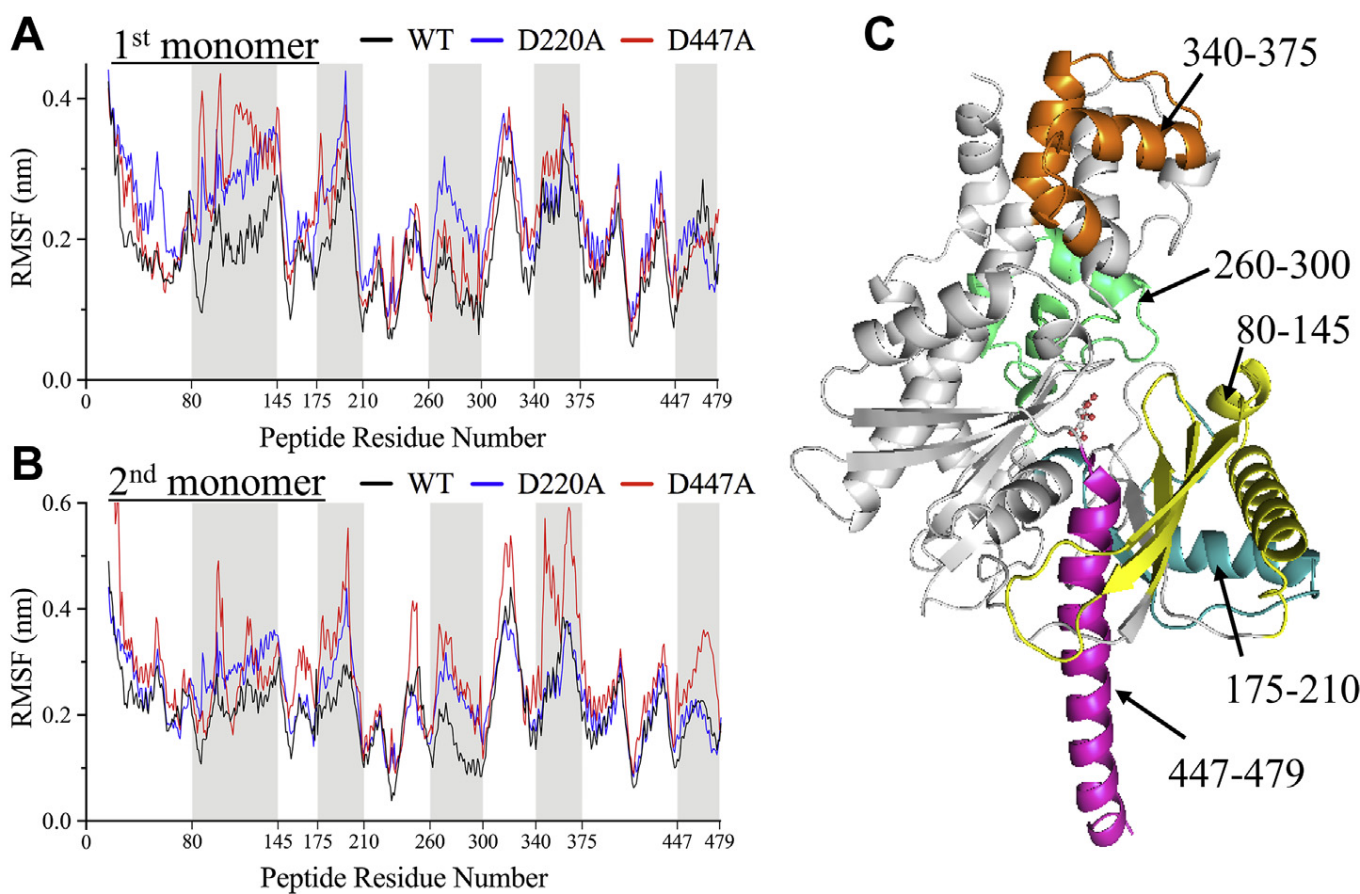
**MD simulation of WT and D447A in the NTD of HK2.***A* and *B*, comparison of PCA-derived RMSF profiles for the first and second monomers of the NTD of HK2 for WT (*black*) and D447A (*red*). *C*, depiction of regions of the 3D structure of the NTD of HK2 that show higher fluctuation in the D447A mutant.

MD simulations at 200 ns were conducted to explore the structural dynamics and fluctuation of the HK2 structure upon the introduction of D447A. For this purpose, principal component analysis (PCA)-derived root mean-square fluctuations (RMSF) were calculated for the C_α_ atoms of WT and D447A (Fig. 7). The first three vectors are presented for each monomer separately and collectively captured >50% of the overall global motion. In the apo-state, the MD simulation trajectories showed increased dynamics of the D447A mutant compared with the WT at the linker helix-α_13_ in addition to regions of the small and large subdomains of the NTD, which was evident from the significant deviation in the PCA-derived RMSF patterns (Fig. 7). The enhanced dynamics observed for the D447A mutant are predicted to be caused by disruption of the interaction between D447 and K451, which has been shown here to be important for NTD activity.

In good agreement with the HDX-MS data, the MD simulation revealed high fluctuation at residues 80–145 and 175–200 of the small subdomain of the NTD (Fig. 7). Similarly, enhanced HDX exchange was also observed at residues 91–107, 127–134, and 189–203 of the small subdomain (Fig. 6). Similar to HDX analysis, the linker helix-α_13_ represented by residues 447–479 also showed slightly higher fluctuation on one of the monomers. Furthermore, high fluctuation was observed in the large subdomain of the NTD at residues 260–300 and 340–375 but was not detected in the HDX-MS analysis. The enhanced dynamics observed in the MD simulation of the D447A mutant compared with WT is due to disruption of the electrostatic interaction of D447 with K451, as mutation at either residue has been shown here to eliminate the NTD activity of HK2. Consequently, the D447A mutation caused displacement of the small and large subdomains and the linker helix-α_13_ from their original positions in the WT (Fig. S7).

The MD simulation has also been conducted on the D220A mutant that increased the NTD activity by 2.5-fold. The dynamics of NTD increased in the presence of D220A mutation compared with the WT, which was expected due to the disruption of its ionic interactions with K451 of the linker helix-α_13_ (Fig. 7). However, D220A was less dynamic than D447A at the small subdomain and linker helix-α_13_ (Fig. S7, *A* and *C*).

Since the active site and linker helix-α_13_ lie in a cleft between the small and large subdomains, the enhanced conformational dynamics induced by the D447A mutation caused wider opening of the NTD active site (Fig. S8*A*). The wider opening of the active site facilitated by D447A is indicated by the large distance between the C_α_ atoms of T88 and T232 of the small and large subdomains of the NTD, respectively (Fig. S8*A*). The distance between T88 and T232 increased from a range of 5 Å–14 Å for the WT to 7 Å–20 Å for D447A. In the case of D220A, the distance was larger compared with the WT, but its distribution was smaller than D447A. In addition, the displacement of the linker helix-α_13_ impacted the relative orientation of the NTD and CTD in the D447A mutant, as represented by the wider angle between the two domains (Fig. S8*B*). The angle was measured at the C_α_ atoms of T389, R468, and A839 on the NTD, linker helix-α_13_, and CTD, respectively. The angle distribution was wider for D447A, with values of 160°–190°, than for the WT, with values of 150°–180° (Fig. S8*B*). Interestingly, a pronounced stabilization effect on the linker helix-α_13_ was observed in the presence of D220A mutation with angle smaller than the WT enzyme (Fig. S8*B*). The enhanced fluctuation and increased opening of the active site upon the introduction of D447A to the NTD could explain the ability of D447 and its interacting partner K451 to eliminate the catalytic activity of the NTD of HK2. The enhanced fluctuation of the NTD in the presence of the D447A mutant could reduce its substrate binding stability, which explains the ability of D447 to eliminate the catalytic activity of the NTD of HK2. On the other hand, the stabilization effect of D220A on the linker helix-α_13_ can explain its ability to increase the NTD activity, which again highlights the importance of the linker helix-α_13_ in the activity of the NTD of HK2.

## Conclusion

Glucose is an essential carbon source for many cancerous tumors, as rapidly proliferating cancer cells alter their metabolic activities to elevate the rate of aerobic glycolysis in order to sustain the high energy and metabolite demands of cancer (1, 3, 4, 5). The HK reaction is an important step for the regulation of glucose metabolism. The upregulation of HK2 in cancer is a major contributor to the elevated aerobic glycolysis and “Warburg effect” in cancer, and the low expression of HK2 in specific normal tissues makes it an attractive target for the selective inhibition of glucose metabolism in cancer (5, 8, 15, 17, 18). The challenge lies in the development of HK2 inhibitors that target cancer but induce limited interference with the ubiquitously expressed HK1 isozyme. Because of the highly conserved structural similarity and active site identity of HK isozymes, the design of inhibitors that target HK2 with limited interactions with the other human isozymes is currently unattainable.

Here, the catalytic roles of 20 amino acid residues of the linker helix-α_13_ and their network of interactions were investigated to determine their effects on the NTD activity of HK2. Three nonactive site amino acid residues (D447, S449, and K451) were found to eliminate the catalytic activity of the NTD of HK2. These amino acid residues are important to the conformational stability of the NTD active site, and alanine substitutions at D447 and K451 increased the structural dynamics of regions surrounding the catalytic helix-α_5_ of the NTD of HK2. HDX-MS analysis and MD simulation of D447A showed higher dynamics and a widened active site cleft in the NTD. The high fluctuation could contribute to reduced glucose binding stability and consequently to poor alignment of glucose with the catalytic residue D209 of the NTD. Disrupting the bonding interactions facilitated by D447, S449, and K451 eliminated the catalytic activity of the NTD of HK2, and thus, these residues represent the newly identified regulatory site of the NTD. A similar pocket also exists in the CTD but was found to be unnecessary for the CTD activity of HK1 and HK2. Since HK2 is the only isozyme with an active NTD among the human HKs, inhibitors that bind and disrupt the interactions in the NTD regulatory site should reduce the activity of HK2 without affecting the activity of HK in normal tissues.

In this study, multiple mutants were identified to increase the activity of the NTD of HK2, in particular, D220A that has the highest catalytic activity compared with the WT. MD simulation confirmed higher stability at the linker helix-α_13_ that may contribute to the increased activity of the NTD for D220A mutant. One of the future objectives of the work presented here is to use structure-based drug design to screen for small molecules that bind the NTD regulatory site of HK2. The inhibition of the NTD will reduce the activity of HK2 and make it similar to other HK isozymes found in normal cells with inactive NTD, *e.g.,* HK1. Targeting the NTD regulatory site will enable exploration of new families of potential HK2 inhibitors and could lead to design and development of novel and safe therapeutic interventions for the treatment of multiple cancer types.

In contrast to residues in the regulatory site, alanine substitutions at T216 and D220, among others, increased the activity of the NTD of HK2. This suggests that these residues enforce structural restraints that stabilize a conformation of the NTD with low catalytic activity. Upon alanine substitutions; however, these restraints are removed and the NTD exhibited higher catalytic activity compared with the WT enzyme. Therefore, these sites can also be targeted in the structure-based drug design of small molecules that can further stabilize these structural restraints, hence reduce the enzymatic activity of the NTD of HK2.

## Materials and methods

### Expression and purification of recombinant mutants human HK2

The recombinant mutants of human HK1 and HK2 were introduced by GenScript Inc into pET28b bacterial expression vector. The expression of the Hisx6-tagged human HK proteins was performed in *Escherichia coli* BL21-CodonPlus-RIL (Stratagene). The inoculated cultures (4–6 l) were grown in LB broth at 25 °C until the A_600_ reached 0.2 in the presence of 100 mg/l kanamycin and 50 mg/l chloramphenicol. The temperature was then lowered to 15 °C and the expression was induced overnight with 0.1 mM IPTG. The cells were harvested by centrifugation at 12,000*g* at 4 °C for 10 min in an Avanti J26-XPI centrifuge (Beckman Coulter Inc), then resuspended in lysis buffer (100 mM Tris pH 7.4, 150 mM NaCl, 5 mM imidazole, 3 mM βME, and 0.1% protease inhibitor cocktail from Sigma-Aldrich: P8849). Cell lysis was carried out using sonication on ice, then centrifuged at 40,000*g* for 45 min at 4 °C. The supernatant was loaded on a ProBond Nickel-Chelating Resin (Life Technologies) previously equilibrated with binding buffer (100 mM Tris pH 7.4, 150 mM NaCl, 5 mM imidazole, and 3 mM βME) at 4 °C. The resin was washed with ten column volumes (cv) of binding buffer, followed by 15 cv of washing buffer (100 mM Tris pH 7.4, 150 mM NaCl, 25 mM imidazole, and 3 mM βME). The His-tagged HK enzyme was eluted from the column with elution buffer (100 mM Tris, pH 7.4, 150 mM NaCl, 300 mM imidazole, and 3 mM βME) in 1 ml aliquots. Finally, the Ni-column fractions containing HK were loaded onto a HiLoad Superdex 200 size-exclusion column using an AKTA purifier core system (GE Healthcare). The column was pre-equilibrated with filtration buffer (50 mM Hepes pH 7.4, 150 mM NaCl, and 0.5 mM TCEP). The final protein was collected and concentrated to ∼5 mg/ml based on Bradford assay. The sample purity was assessed using SDS-PAGE (Fig. S1).

### Enzyme assay and initial velocity studies

HK activity was determined by the glucose-6-phosphate dehydrogenase coupled spectrometric assay as described (37) using Shimadzu UV-visible Spectrophotometer (UV-2700) equipped with CPS-100 temperature controller. The reaction was monitored at 340 nm (ε_340nm_ = 6220 M^−1^ cm^−1^) in an enzymatic reaction containing 50 mM Hepes pH 7.4, 150 mM NaCl, 20 mM MgCl_2_, 3 mM NAD^+^, and 0.1 U/μl G6PDH (Sigma- 8404). The initial linear portion of the time course was fitted to a straight-line function to calculate the initial velocity using the Excel add-on package XL fit (IDBS Limited). The enzyme titration assay was conducted by measuring the enzyme rate at fixed 3 mM concentration of glucose and ATP, and the enzyme concentration was varied from 0.05 to 0.5 μM, and the initial rate (*v*) was plotted against the enzyme concentration. The enzyme rate was fitted to a straight-line equation, and the relative enzymatic activity was calculated from the slope of the line. The graphs were created using the GraphPad Prism 6 software (GraphPad Software, Inc).

The initial enzyme rate in the direction of G6P formation was measured as a function of glucose concentration (0.025 mM–2 mM) at different fixed concentrations of ATP (0.05–3 mM). The amount of enzyme used in the reaction was kept constant at 65 nM for the wild-type HK2, but varied for the mutants relative to their activity. The initial rate data was plotted in a double reciprocal form to determine the quality of the data, then fitted using Equation 1 for a random Bi–Bi sequential kinetic mechanism.

(1) $$v\;=\;\frac{V_{\text{max}}AB}{K_{\text{a}}K_{\text{b}}\;+\;K_{\text{b}}A\;+\;K_{\text{a}}B\;+\;AB}$$

where *v* and *V*_max_ are the initial and maximum velocities, *A* and *B* are the substrate concentrations, and *K*_a_ and *K*_b_ are the Michaelis constants for substrates *A* and *B*, respectively. Data were globally fitted using the kinetics module of SigmaPlot (Systat Software, Inc).

### Thermal inactivation of HK2 enzyme

Thermal inactivation kinetics of HK2 at 37 °C in the presence or absence of 3 mM glucose were analyzed using two-state mechanism (N↔U). The enzyme was incubated at 37 °C, then its activity was measured at different incubation time intervals. The enzyme rate was assayed spectroscopically and the % residual enzyme activity was plotted as a function of HK2 incubation time. Thermal inactivation was analyzed considering first-order activity decay, and the enzymatic activity decay was fitted to a first-order reaction, Equation 2 or to a signal phase exponential decay using Prism 6, GraphPad Software.

(2) $$ln\left[A\right]\;=\;-k_{obs}t\;+\;ln\left[A_{0}\right]$$

where *A*_0_ and *A* are the initial and remaining enzyme activity at different time intervals, respectively. *k*_*obs*_ is the rate constant for enzyme inactivation, and *t* is the incubation time. The half-life (*t*_1/2_) is the time required to decrease the enzyme activity by half, was calculated as ln(2)/*k*_*obs*_.

### Differential scanning calorimetry and differential scanning fluorimetry

The thermodynamic stability of the HK enzymes was measured using Nano-DSC (TA Instruments). The thermogram was measured in the absence or presence of 5 mM glucose using 5 μM enzyme. The buffer (50 mM Hepes pH 7.4, and 150 mM NaCl) was degassed under vacuum for 15 min. The total volume of 700 μl was loaded into the sample cell, and the reference cell that contains all components except the enzyme sample. The sample was heated at a scan rate of 1 °C/min from 10 to 80 °C at 3 atm pressure. The background scans were obtained by loading degassed buffer (with or without glucose) in both the reference and samples cells and heated at the same rate. A reheating scan was carried out on all samples to establish the irreversibility of the thermal denature of HK2 samples. In addition to the background scan, the reheating scan was used as a blank. The DSC thermograms were corrected by subtracting the corresponding buffer baseline and converted to plots of excess heat capacity (*C*_p_) as a function of temperature. The Nano-Analyze software package from TA instruments was used for baseline subtraction and deconvolution of the DSC thermograms. The melting point (T_m_) is equal to the maximum temperature of the thermal transition, and the calorimetric enthalpy of the transitions (Δ*H*_cal_) was estimated by calculating the area under the thermal transition.

The thermal T_m_ of the HK enzyme was determined using DSF measurements in the presence of SYPRO Orange fluorescent dye using a real-time QPCR instrument (Mx3005P QPCR system, Agilent Technologies). The measurements were conducted in a 96-well thin-walled PCR microplate (BioRad, Cat. No. 223 94444) with excitation and emission at 492 nm and 610 nm, respectively. The protein sample contains 50 mM Hepes pH 7.4, 150 mM NaCl, and 40 mM MgCl_2_ in the presence of 5 μM enzyme and 3X SYPRO Orange dye. The concentration of the substrates, ATP, and glucose were varied at 0 mM–10 mM. The fluorescence measurements of the protein unfolding signals were collected from 25 °C to 80 °C at a temperature ramp rate of 1 °C/min. The data was fitted to a Boltzmann sigmoidal function to calculate the T_m_ at the middle of the transition using the Excel add-on package XLfit (IDBS limited) as described previously (38).

### Hydrogen/deuterium exchange mass spectrometry

The HDX reaction was initiated by diluting 3 μl of either 100 μM NTD or FL-variants into 37 μl or 97 μl, respectively, of D_2_O (Sigma-Aldrich) buffer (20 mM HEPES pD 7.5, 150 mM NaCl, and 0.5 mM TCEP). Since the FL variant was approximately twice the molecular weight of the NTD, a 2.5 dilution factor for FL was used compared with NTD to avoid overloading the protease column with final injection concentrations of 7.5 μM and 3 μM for NTD and FL-variants, respectively. The exchange reaction was performed at 25 °C with different time points (1 min, 6 min, 1 h, and 3 h). However, due to low HDX rates, the exchange reaction incubation temperature was increased to 37 °C in order to increase the protein dynamics and deuterium exchange rate. Each time point was repeated between four and eight times in the presence or absence of 5 mM glucose. The exchange reaction was quenched at 4 °C with 1:1 dilution in quench buffer for NTD (100 mM Phosphate pH 2.2, 2 M Guanidine-HCl, and 200 mM TCP) and FL-variant (2 M Guanidine-HCl, 0.8% formic acid pH 2.2). The nondeuterated experiments were performed under the same conditions and quench buffer. A volume of 60 μl or 185 μl of the final reaction mixture for the NTD or FL-variant, respectively, was injected on the online protease and liquid chromatography mass spectrometry. The HDX PAL RTC Robot (LEAP Technologies) with refrigerated sample compartments was used to automate the HDX reactions and injections into the mass-spectrometer.

### LC-MS and MS/MS analysis

An isocratic pump (MX-Class; Teledyne SSI) with 100 μl/min of 0.3% formic acid was used to load the reaction mixture onto the online immobilized pepsin column (2.1 × 30 mm; Waters). The column was kept at 4 °C and NTD and FL-variants were digested at 4 min and 6 min, respectively. A gradient pump (1290 Infinity; Agilent Technologies) with C18 Trap column (1.7 μm × 30 mm; Waters) and analytical columns (1.7 μm, 1.0 × 100 mm; Waters) were used to desalt and separate generated peptides. Both C18 columns were kept at 4 °C at constant flow rate of 40 μl/min. The mobile phases consisted of aqueous 0.3% formic acid (Sigma-Aldrich) for solvent A and 0.3% formic acid in 95% acetonitrile for solvent B. A linear gradient was set at 10% to 40% of solvent B in 10 min, then to 70% of solvent B in 5 min. A 10 min wash at 95% of solvent B was used to maintain the column sensitive and prevent carry over followed by 15 min equilibration with 10% of solvent B to complete the gradient. The LC system was coupled to a QTOF Impact II mass spectrometer (Bruker Daltonik) equipped with an Easy Spray ion source and operated in positive ion mode. The spray voltage was set to 4.5 kV, dry gas 4.0 l/min, dry temperature 100 °C, and the full scans were acquired in a TOF MS mass analyzer over *m/z* 300–2000 at a spectra rate of 2.0 Hz.

For protein sequence coverage and peptide identification, the auto MS/MS analysis with a fixed precursor cycle time of 2 s was performed using collision induced dissociation (CID). The MS/MS was performed only for the nondeuterated sample. The MS/MS raw files, converted to mgf format by DataAnalysis software (Bruker Daltonik) with 622 *m/z* lock mass calibration, were searched against HK2 sequence using the ProteinScape software (Bruker Daltonik) with an in-house Mascot search engine (Matrix Science Limited). The search parameters were set at peptide tolerance of 10 ppm, MS/MS tolerance of 0.05 Da, mascot score of 20 for positive peptide identification, and oxidation of methionine and protein N-terminal acetylation were used as variable modifications. The peptide deuterium-exchange data were calculated as the change in mass of deuterated and nondeuterated averaged masses of the protein using HDExaminer (Sierra Analytics). HDExaminer performs automatic isotopic envelope isolation and measures the average mass of peptides. The statistical analysis for deuterium labeling on the peptide was further analyzed by GraphPad Prism 6 software (GraphPad Software, Inc).

### 3D structure preparation for molecular dynamics simulations

The crystal structure of human HK2 in the dimeric form (PDB-ID: 2NZT) was used in the MD simulations (12). Prior to structure preparation, glucose and G6P were removed from the crystal structure to acquire the apo-state of HK2. The missing parts of the protein were modeled using “Crosslink Protein” panel of BioLuminate, which is implemented in Schrodinger suite (39). The hydrogen atoms were added using “Protein preparation” wizard of Schrodinger suite (40). The protonation states of amino acids were determined using PROPKA at pH 7.0 (41). The D447A mutant of the NTD was introduced using the “Build” panel of Schrodinger suite (40).

### The MD simulation setup

The MD simulation files were prepared using CHARMM-GUI web server (42). The protein was modeled by CHARMM36 force field, whereas water molecules were modeled using TIP3P model (43, 44). The systems were neutralized with salt concentration of 0.15 M KCl, and simulations were carried out using GROMACS software (45). Periodic boundary conditions were applied in all directions and simulation time step was set to 2 fs. The temperature and pressure were maintained at 310 K and 1 atm using Nose–Hoover and Parrinello–Rahman coupling algorithms, respectively to achieve canonical ensemble (46, 47, 48). Particle mesh Ewald (PME) method was used to compute long-range electrostatic forces and Van der Waals forces were treated with 9 Å cutoff (49). The systems were simulated for 200 ns, where the backbone RMSD of the protein fluctuated no more than 2 Å. All coordinates were saved at 10 ps intervals for further analysis. Two separate simulations were performed, where each was started with different initial velocity distribution.

### Analysis of the MD simulation trajectories

PCA was carried out on the apo-state of the wild-type and mutant proteins to explore dominant motions in the systems. First, the resultant trajectories of each system were aligned with respect to backbone C_α_ atom of the corresponding initial structure. Computation and diagonalization of covariance matrices were done by using “gmx covar” module of GROMACS, and the “gmx anaeig” module of GROMACS was used to obtain eigenvectors and eigenvalues from diagonalized covariance matrices (45). The first three vectors, which collectively captured more than 50% of the overall global motion, were presented for each monomer separately. The distance measurement was done using the C_α_ atoms of T88 and T232, which are located in the small- and large-subdomains of the NTD, respectively (Fig. S8). On the other hand, the angle measurement was done using the C_α_ atoms of T389, R468, and A839 of the NTD, linker helix-α_13_, and CTD, respectively (Fig. S8).

## Data availability

All data are included within the article and associated Supporting information.

## Conflict of interest

The authors declare that they have no conflicts of interest with the contents of this article.
