# Supplementary material for: Linker residues regulate the activity and stability of hexokinase 2, a promising anticancer target
Source: J Biol Chem. 2020 Nov 24;296:100071. doi: 10.1074/jbc.RA120.015293 (PMC7949118; doi:10.1074/jbc.RA120.015293)
Supplement: Figures S1 to S7 [file mmc1.pdf]

Supporting information

Linker residues regulate the activity and stability of hexokinase 2, a promising anticancer target

Juliana C. Ferreira[1], Abdul-Rahman Khrbtli[1], Cameron Lee Shetler[2], Samman Mansoor[3], Liaqat Ali[4], Ozge Sensoy[3,5,6], Wael M. Rabeh[1*]

[1]Science Division, New York University Abu Dhabi, PO Box 129188, Abu Dhabi, United Arab Emirates
[2]Department of Chemistry, New York University Shanghai, 1555 Century Avenue, Shanghai 200122, China
[3]The School of Engineering and Natural Sciences, Istanbul Medipol University, 34810 Istanbul, Turkey
[4]Core Technology Platforms, New York University Abu Dhabi, Saadiyat Campus, P.O. Box 127788, Abu Dhabi, UAE
[5]Regenerative and Restorative Medicine Research Center (REMER), Istanbul Medipol University, 34810, Istanbul, Turkey
[6]Research Institute for Health Sciences and Technologies (SABITA), İstanbul Medipol University, 34810, Istanbul, Turkey

[*] Corresponding author: Wael M. Rabeh
E-mail:wael.rabeh@nyu.edu

Content:

Supporting figures S1-S8

Supporting figures

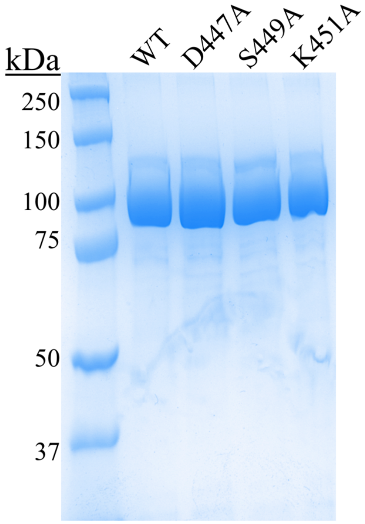

Figure S1: SDS-PAGE analysis of human HK2 variants. The purity of WT and mutant HK2 proteins was assessed using SDS-PAGE. The lanes were labeled according to each variant and a molecular weight (MW) marker was included in the first lane.

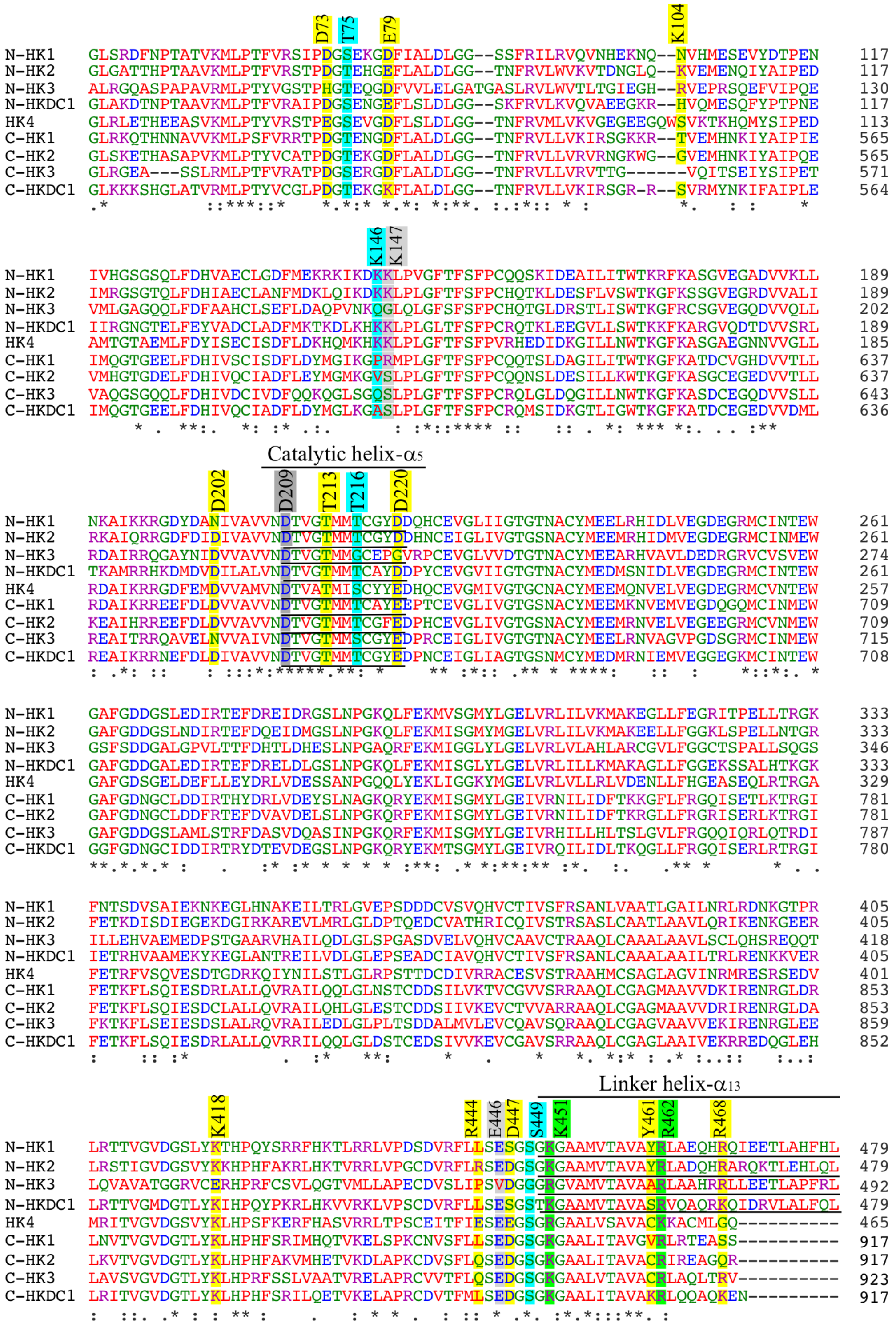

Figure S2: Amino acid sequence alignment of human hexokinase isozymes. Amino acid sequence alignment of the individual NTD and CTD of the human isozymes HK1, HK2, HK3, and HKDC1 in addition to the single domain HK4 was carried out using Clustal Omega at EMBL[47]. The residue numbers listed above the amino acid sequences are for the NTD of HK2. Residues with shaded backgrounds are the mutation sites introduced here into HK2. The residues of the catalytic helix-α_5 and linker helix-α_{13} are underlined.

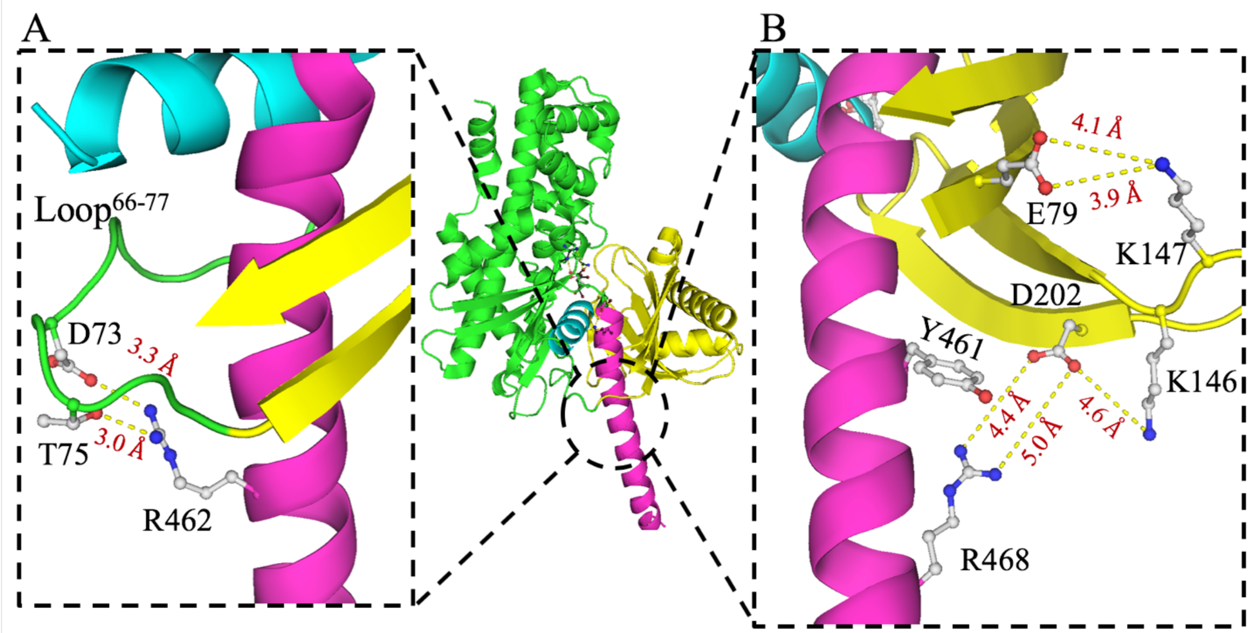

Figure S3: Molecular interactions of the linker helix-α_{13} with loop$^{66-77}$ and the small subdomain. (**A**) The small subdomain is connected to the large subdomain by loop$^{66-77}$ (green) and the catalytic helix-α_5 (cyan). The color code is similar to that in Fig. 1. D73 and T75 of loop$^{66-77}$ form ionic (3.3 Å) and hydrogen bond (3.0 Å) interactions with R462 of the linker helix-α_{13}, respectively. (**B**) The side chain of R468 on the linker helix-α_{13} is capable of forming ionic interactions with D202 at 4.4 Å. In the small subdomain, ionic interactions can also be formed between E79 and K147 (3.9 Å) and K146 and D202 (4.6 Å). The catalytic activity of Y461 in the linker helix-α_{13} was also investigated. This figure was prepared using PyMol (Schrodinger LLC).

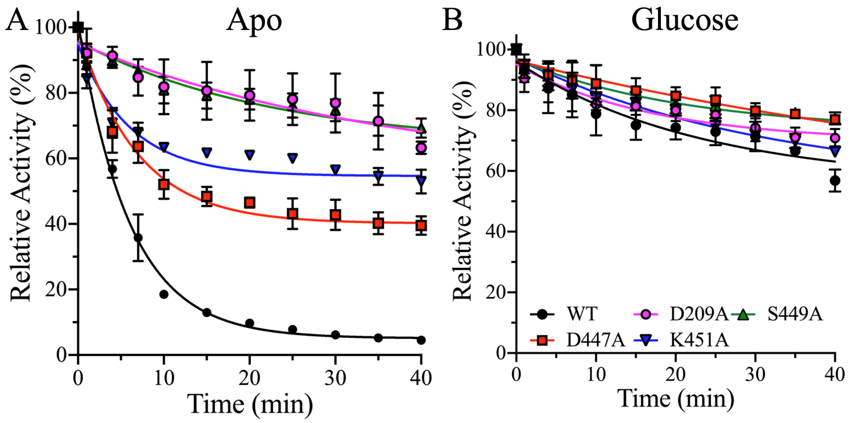

Figure S4: Heat inactivation kinetics of NTD mutants. The residual enzymatic activity of the CTD was determined at different time intervals in the FL variant and in the presence of mutants that inactivated the NTD of HK2. The enzyme was incubated in the absence (**A**) or presence of glucose (**B**) at 37 °C for the WT and NTD mutants of HK2. Data are the mean ± S.D., n= 3.

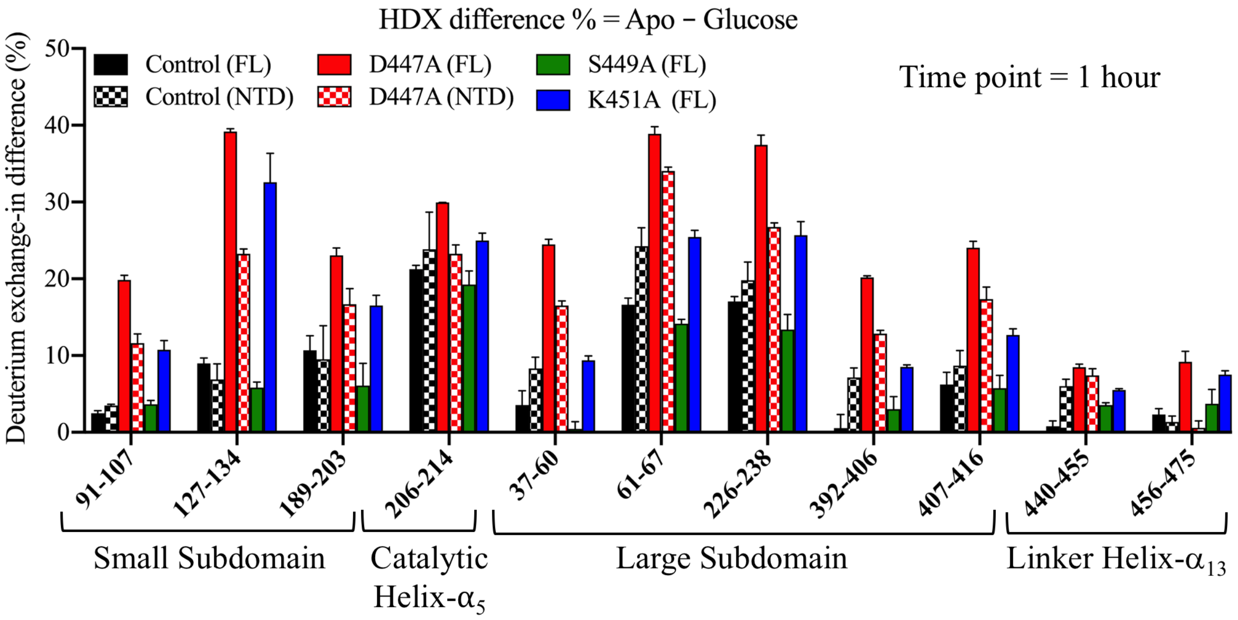

Figure S5: Comparison of HDX protection by glucose for the WT and NTD mutants of HK2. The percent increase in deuterium exchange difference of the apo-state at 1 h compared to the glucose-bound state for WT (black) and D447A (red), S449A (green), and K451A (blue) in FL (solid bars) and NTD (checkered bars) variants. The percent increase in deuterium exchange was calculated by subtracting the percentage of deuterium incorporation for the glucose-bound state from the apo-state. The highest deuterium exchange difference between the apo-state and glucose-bound states was observed for D447A compared to other mutants and the WT, while S449A had the lowest difference. Overall, peptides 206–214 of the catalytic helix-α_5 and 61–67 and 226–238 of the large subdomain showed the highest difference in deuterium exchange between the apo and glucose-bound states. On the other hand, peptides 440–455 and 456–475 of the linker helix-α_{13} showed no difference in HDX rate for WT and a slight decrease in deuterium exchange for the mutants upon the addition of glucose.

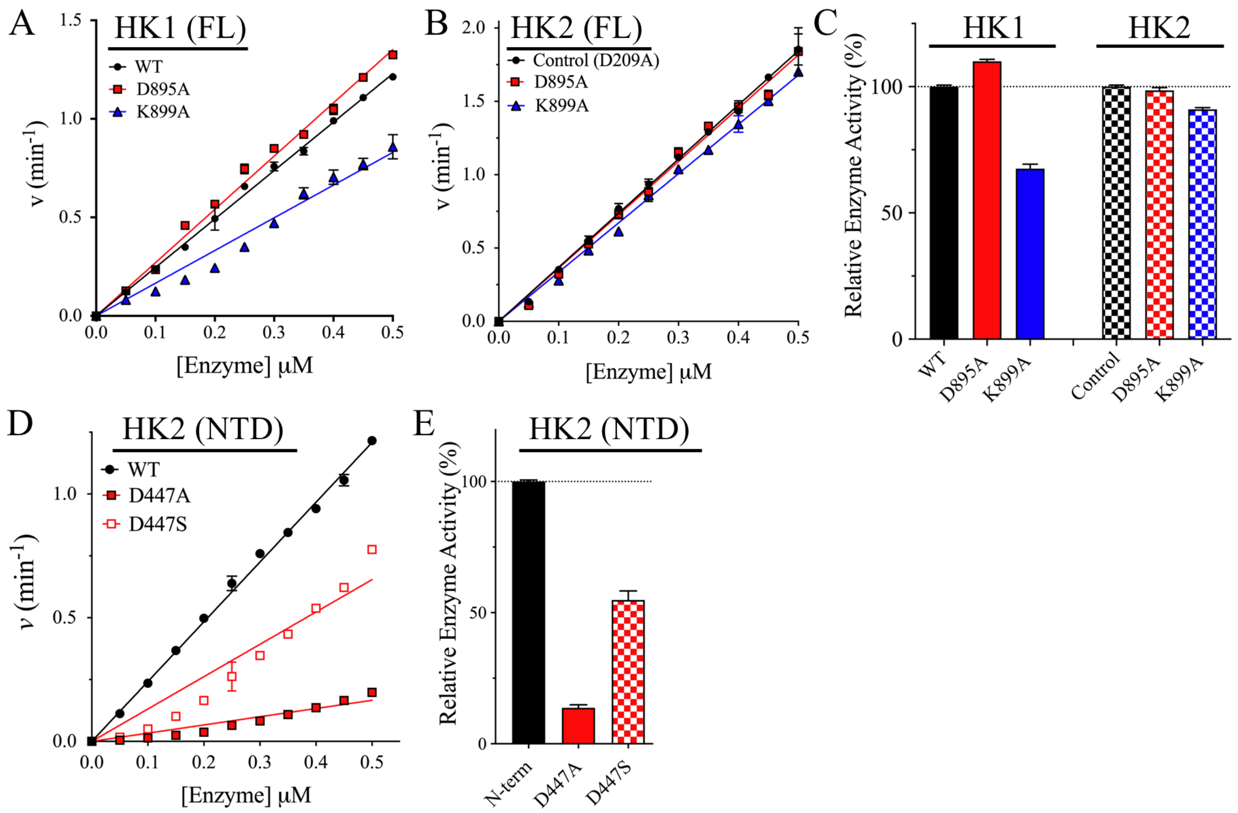

Figure S6: Enzyme titration analysis of the CTD mutants of HK1 and HK2 in the FL variant and NTD mutants of HK2 in the NTD variant. (A-B) The enzyme rates of CTD mutants of HK1 and HK2 in the FL variant measured at different enzyme concentrations with fixed saturated concentrations of 3 mM for glucose and ATP. For the HK2 enzyme, the CTD activity was measured directly in the FL variant in the presence of D209A mutants to inactivate its NTD. The HK1 enzyme has an inactive NTD, and the D209A mutation was not needed. **(C)** Bar plot of relative enzyme activity of the CTD mutants that was determined from the slope of the enzyme rates. The CTD activity was unaffected or slightly changed in the presence of D895A and K899A in the HK1 and HK2. **(D)** Enzymatic rates of WT and D447 mutants in the NTD variant of HK2 that were measured as in panel A. (**E)** Bar plot of relative enzyme activity of the NTD mutant of HK2 in the NTD variant, determined from the slope of enzyme rates. The enzymatic rates of D447A and D447S were 14% and 55% of the WT, respectively. Data are the mean ± S.D., n=3.

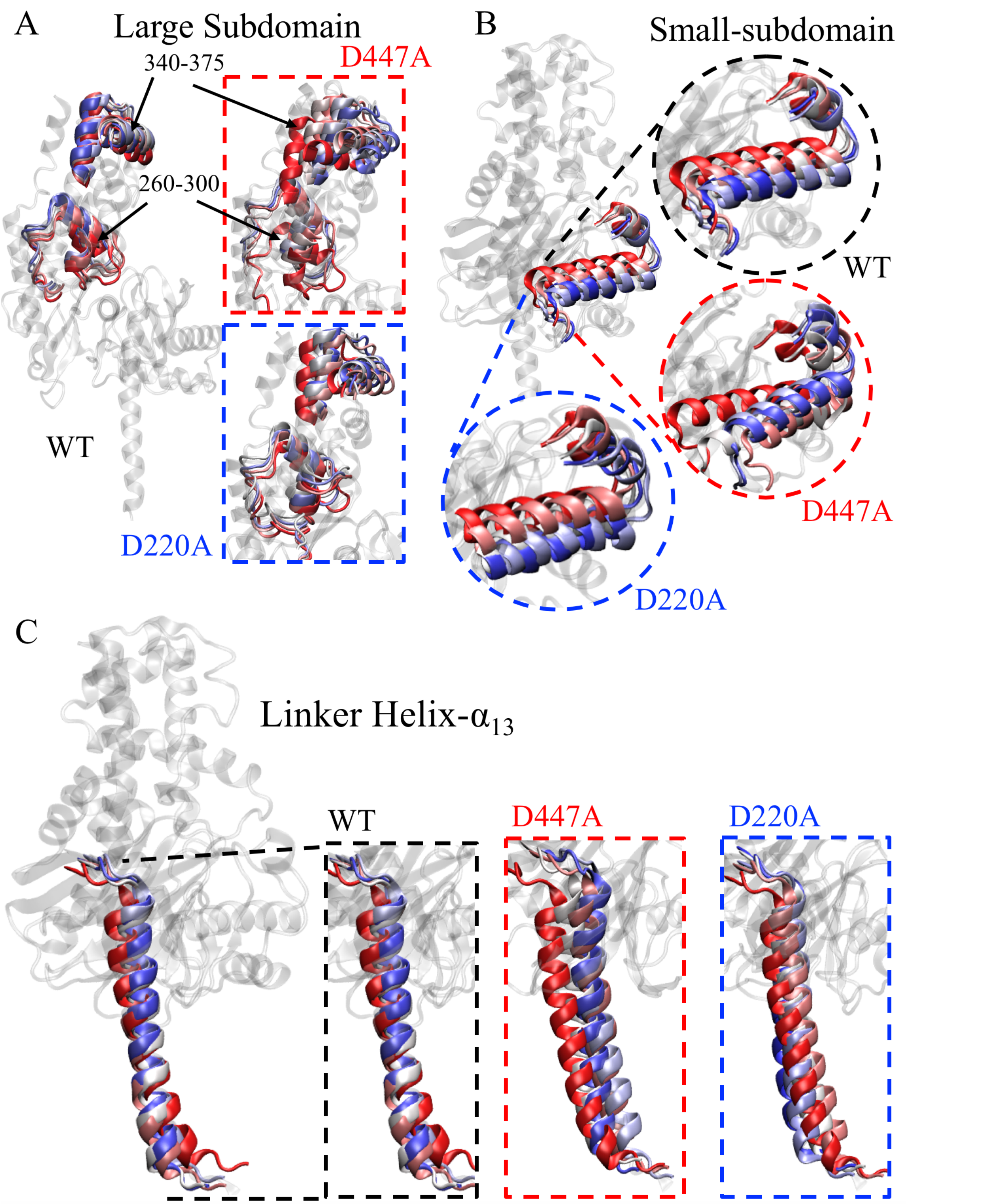

Figure S7: Enhanced dynamics of D447A mutant in the NTD of HK2. Comparative depiction of NTD regions with enhanced dynamics in D447A compared to WT for the small subdomain (**A**), large subdomain (**B**), and linker helix-α_{13} (**C**).

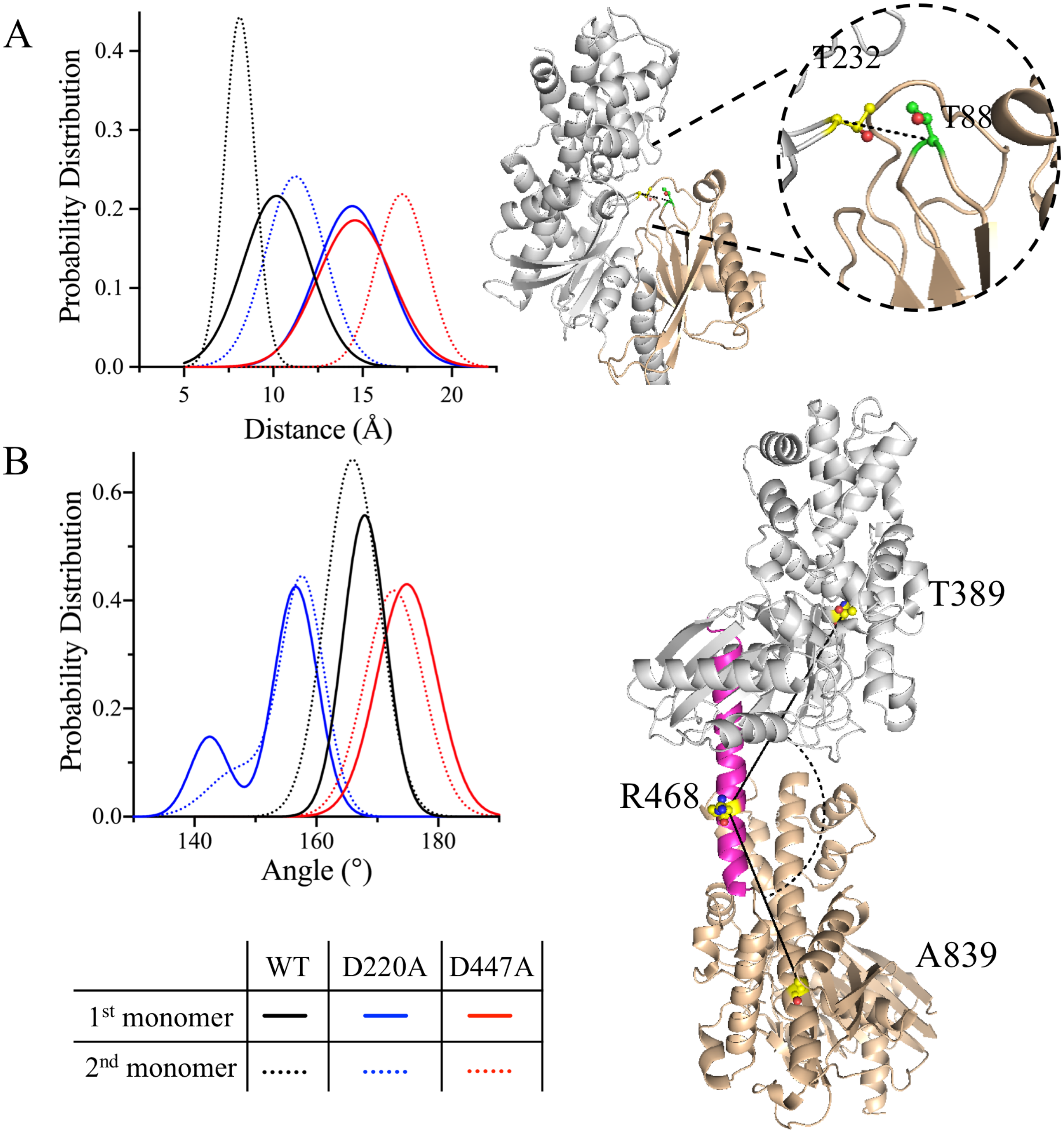

Figure S8: Distance and angle probability distributions of WT and D447A. (**A**) Distance probability distribution calculated between C_{α} atoms of residues T88 and T232 displayed in a sphere representation. (**B**) Angle probability distribution calculated between $C_{\square}$ atoms of residues T389, R468 and A839, shown as spheres.
